# Inflammatory macrophage-derived plasminogen activator inhibitor-1 exacerbates inflammation through efferocytosis inhibition

**DOI:** 10.1038/s41420-026-03076-0

**Published:** 2026-03-27

**Authors:** Abd Aziz Ibrahim, Hiromi Miura, Tomoya Terada, Masaki Kawarada, Nobuo Watanabe, Hiroyuki Hosokawa, Masato Ohtsuka, Toshio Miyata, Takashi Yahata

**Affiliations:** 1 Translational Molecular Therapeutics Laboratory, Division of Host Defense Mechanism, Tokai University School of Medicine, Isehara, Kanagawa Japan; 2 Department of Molecular Life Science, Division of Basic Medical Science and Molecular Medicine, Tokai University School of Medicine, Isehara, Kanagawa Japan; 3 Department of Emergency and Critical Care Medicine, Tokai University School of Medicine, Isehara, Kanagawa Japan; 4 Department of Immunology, Division of Host Defense Mechanism, Tokai University School of Medicine, Isehara, Kanagawa Japan; 5 The Institute of Medical Sciences, Tokai University School of Medicine, Isehara, Kanagawa Japan; 6 Department of Molecular Medicine and Therapy, United Centers for Advanced Research and Translational Medicine, Tohoku University Graduate School of Medicine, Sendai, Miyagi Japan

**Keywords:** Chronic inflammation, Cell death and immune response

## Abstract

Plasminogen activator inhibitor-1 (PAI-1) is significantly upregulated during inflammatory responses, and elevated PAI-1 levels are associated with poor prognosis in various diseases. However, the precise mechanism through which PAI-1 exacerbates inflammation remains unclear. In the present study, we have investigated the role of PAI-1 in inflammation using a mouse model of skeletal muscle injury. We found that CCR2⁺Ly6C⁺ inflammatory macrophages infiltrated the injured tissues and produced substantial amounts of PAI-1. Notably, PAI-1 deficiency specifically in these macrophages resulted in attenuated inflammation and accelerated tissue repair despite the continued presence of PAI-1 in body fluids, indicating a local macrophage-driven effect. Low-density lipoprotein receptor-related protein-1 (LRP-1), expressed on macrophages, is a common receptor for both PAI-1 and calreticulin (CRT). CRT is exposed on the surface of dying cells and functions as an “eat me” signal recognized by macrophages *via* LRP-1. We found that PAI-1 binds to LRP-1 with higher affinity than that to CRT, thereby competitively inhibiting CRT recognition and suppressing efferocytosis, the process by which macrophages clear dead cells, ultimately leading to prolonged inflammation. Importantly, administration of a PAI-1 inhibitor, TM5614, restored efferocytosis and significantly improved tissue regeneration. These findings therefore reveal that PAI-1 produced by infiltrating inflammatory macrophages contributes to sustained inflammation by blocking efferocytosis, and that PAI-1 is a promising therapeutic target for the treatment of inflammatory diseases.

**Figure:**
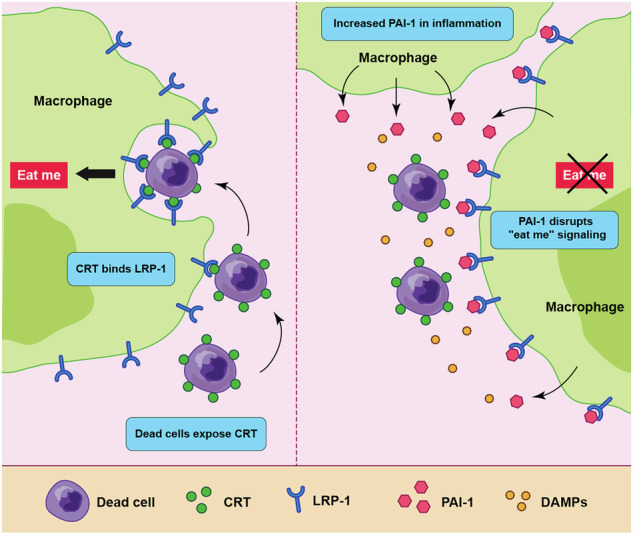
Dead cells expose an “eat-me” signal molecule CRT, which is recognized by the LRP-1 receptor on macrophages, leading to their phagocytosis through a process known as efferocytosis. However, PAI-1, secreted by infiltrating CCR2^+^Ly6c^+^ macrophages, impairs tissue regeneration after injury by inhibiting efferocytosis through competitive binding to LRP-1, thereby prolonging inflammation.

Dead cells expose an “eat-me” signal molecule CRT, which is recognized by the LRP-1 receptor on macrophages, leading to their phagocytosis through a process known as efferocytosis. However, PAI-1, secreted by infiltrating CCR2^+^Ly6c^+^ macrophages, impairs tissue regeneration after injury by inhibiting efferocytosis through competitive binding to LRP-1, thereby prolonging inflammation.

## Introduction

Inflammation is a fundamental defense mechanism in human body that aims to eliminate foreign substances, such as viruses and microbes, as well as damaged or dead cells [1]. However, persistent inflammation can contribute to the onset, progression, and exacerbation of various diseases, including cancer, neurodegenerative disorders, atherosclerotic diseases, and autoimmune diseases, thus establishing a central role for inflammation in the etiology of numerous pathologies [2–5]. Macrophages, a cell population characterized by functional versatility, play a crucial role in regulating various inflammatory conditions by facilitating the resolution of inflammation and promoting tissue repair through efferocytosis, a process for phagocytosing dead cells [6–9]. Disruptions in this seemingly simple “to eat or not to eat” function at the injury or infection sites can significantly affect systemic homeostasis maintenance [10].

The process of efferocytosis, or dead cell ingestion by macrophages, is governed by a delicate balance between “eat me” and “don’t eat me” signals expressed on the surface of the dead cell. The transmission of an apoptotic signal to a cell results in the exposure of calreticulin (CRT), a calcium ion (Ca^2+^)-binding protein located in the endoplasmic reticulum on the outer cell surface. The receptor low-density lipoprotein-related peptide-1 (LRP-1), which is expressed on the macrophage surface, binds to CRT on the surface of dead cells, thereby converting them into “eat me” signals [11, 12]. The subsequent activation of molecules, such as CD14 and CD91, or small GTPases belonging to the Ras and Rho families, on the phagocyte surface facilitates dead cell engulfment by macrophages. These cells are internalized, forming vesicles known as efferosomes, which are then degraded and processed. In contrast, molecules, such as CD47, are expressed on the surface of normal cells and recognized as “don’t eat me” signals, preventing efferocytosis in healthy cells [13, 14].

Macrophages are classified into inflammatory and suppressive macrophages (also known as repairing macrophages), based on the Ly6C molecule expression levels on their surface [6]. In response to tissue damage owing to trauma or infection, circulating monocytes are recruited to the injury site. There, they undergo a process of differentiation into Ly6C^+^ inflammatory macrophages, which subsequently secrete pro-inflammatory cytokines, such as tumor necrosis factor-α (TNF-α). These inflammatory cytokines, in turn, act on the surrounding cells and vascular endothelium, promoting immune cell migration to the inflammation site. Monocyte chemoattractant protein 1 (MCP-1), also known as C–C motif chemokine ligand 2 (CCL2), is produced at the inflammation site and plays a key role in recruiting Ly6C^+^ monocytes/macrophages expressing its receptor, CCR2 (CC chemokine receptor 2), to the inflammation site [15, 16]. Consequently, more immune cells accumulate at the inflamed site, amplifying the local inflammatory response. Macrophages play a crucial role in inflammation resolution. After triggering the inflammatory response, Ly6C^+^ macrophages differentiate into Ly6C^−^ suppressive macrophages, which produce anti-inflammatory cytokines, such as interleukin-10 (IL-10), and promote inflammation resolution by phagocytosing dead cells. This process facilitates normal tissue restoration.

When tissue damage, inflammation, aging, tumorigenesis, or other factors increase damaged or dead cells beyond the normal capacity for efferocytosis, these damaged or dead cells accumulate owing to insufficient removal. A similar outcome was observed in cases with a decline or impairment of efferocytosis. For instance, impaired or reduced efferocytosis function prolongs inflammation, potentially contributing to the progression and exacerbation of chronic inflammatory conditions, such as atherosclerosis, diabetes, heart failure, and malignancies. Dead cell accumulation has been associated with the onset and exacerbation of various diseases as well as the aging process. Efferocytosis enhancement (or improved efferocytosis dysfunction) may suppress damaged or dead cell accumulation, thereby contributing to homeostasis maintenance and the promotion of the early resolution of inflammation. This approach may mitigate or improve age-related diseases, tumor-related conditions, and inflammatory disorders. However, the mechanisms regulating efferocytosis remain poorly understood.

Plasminogen activator inhibitor-1 (PAI-1) plays a crucial role in regulating fibrin degradation in the fibrinolytic system [17]. It is predominantly found in adipocytes, vascular endothelial cells, platelets, and the liver, and contributes to blood coagulation regulation by suppressing the generation of plasmin, which breaks down fibrin. Its expression is induced by inflammatory cytokines, such as TNF-α, and it is upregulated in various inflammatory diseases. PAI-1 is likely to exacerbate inflammation and plays a multifaceted role in muscle diseases and functional impairment. For example, during the repair process following skeletal muscle injury, PAI-1 expression potentially promotes extracellular matrix accumulation and contributes to muscle fibrosis [18–21]. Conditions, such as muscular dystrophies (e.g., Duchenne muscular dystrophy) and chronic myositis, have been linked to PAI-1 overexpression, which may exacerbate fibrosis and muscle weakness. Additionally, PAI-1 expression tends to increase with aging [22, 23], potentially contributing to chronic inflammation, muscle atrophy, impaired regenerative capacity, and reduced muscle mass, as evidenced by ongoing research. These findings suggest a pivotal role for PAI-1 in inflammatory disease progression and persistence. However, the mechanism through which PAI-1 acts as a pathogenic factor in several inflammatory diseases remains unclear.

In this study, we focused on the receptor LRP-1, which recognizes CRT expressed on dead cells and serves as a receptor for PAI-1. We proposed that PAI-1 competitively inhibits the binding of CRT and LRP-1, thereby interfering with “eat me” signal recognition by macrophages. Based on this, we aimed to test the hypothesis that PAI-1 suppresses the efferocytic function of macrophages, thereby reducing dead-cell clearance efficiency, potentially leading to prolonged and exacerbated inflammatory responses. To evaluate this, we used a murine model of skeletal muscle injury-induced inflammation to explore the role of PAI-1 and its therapeutic potential.

## Results

### Injury-induced inflammation triggers PAI-1 upregulation

**Fig. 1:**
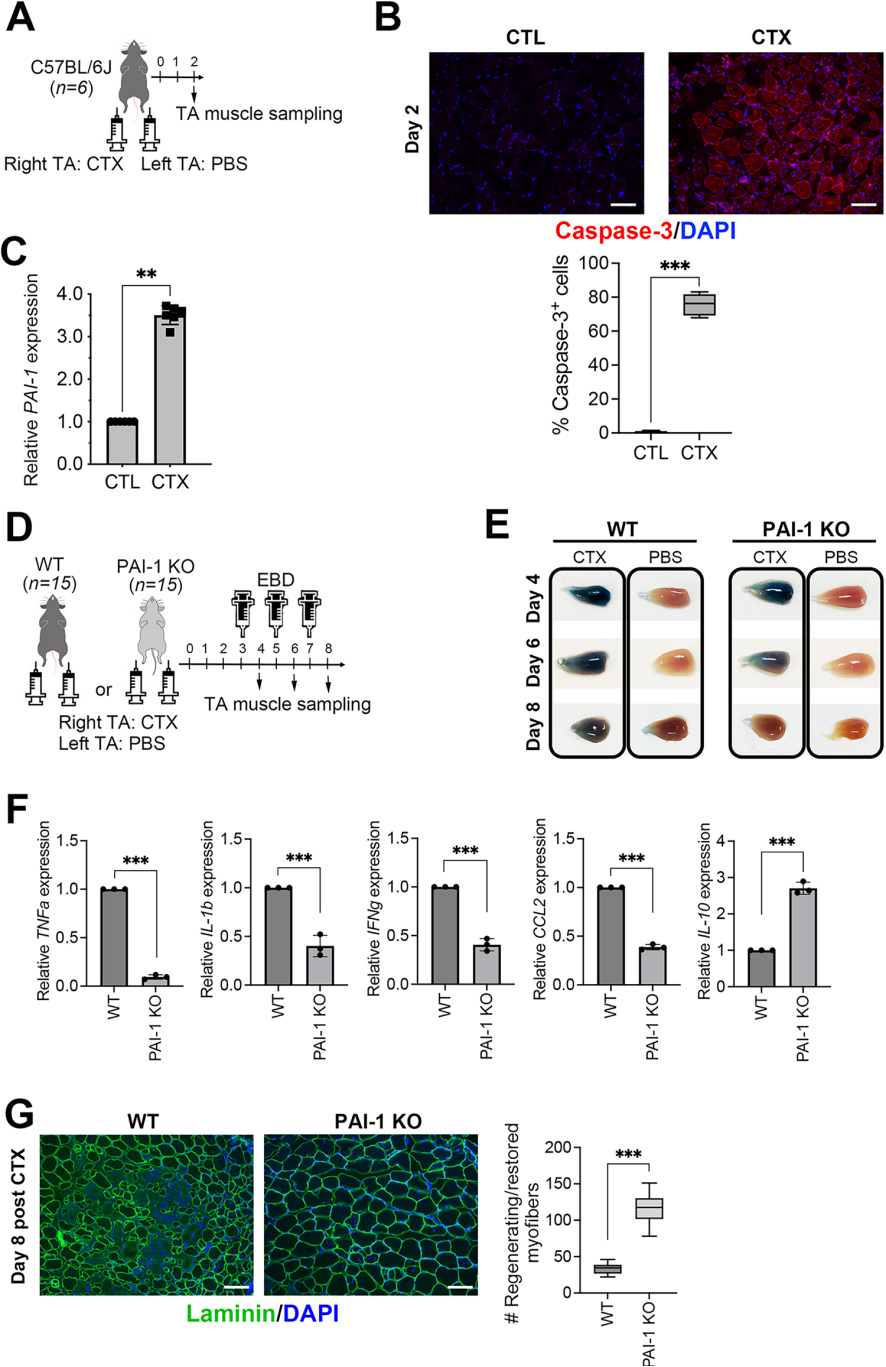
PAI-1 inhibits tissue regeneration. **A** Experimental scheme of CTX-induced TA muscle injury. **B** Representative immunohistochemical images of the TA muscle showing cleaved-Caspase-3 (red) and DAPI (blue) staining in CTX-treated mice at day 2 post-injury. Bars represent 100 μm. The box-and-whisker plot shows the percentage of cleaved Capase-3^+^ cells in the TA muscles. More than 10 sections from randomly selected fields per slide were counted for each of six mice per group. ^***^p < 0.001. **C** PAI-1 mRNA expression after CTX-induced TA muscle injury (*n* = 6/group). The bars express the results as the mean ± SD of three independent experiments. ^**^p < 0.01. **D** Experimental scheme of CTX-induced TA muscle injury in WT and PAI-1 KO mice (*n* = 12/group). EBD was injected intraperitoneally into mice 16 h before TA muscle sampling. **E** Representative images of EBD-stained TA muscle in WT and PAI-1 KO mice on days 4, 6, and 8 post-injury. The original unprocessed images are shown in Supplementary Fig. S3. **F** Pro-inflammatory and anti-inflammatory cytokine mRNA expression in the TA muscles from the WT and PAI-1 KO mice at day 4 after CTX-induced injury (*n* = 3/group), analyzed by qPCR. The bars express the results as the mean ± SD of three independent experiments. ^***^p < 0.001. **G** Representative images of Laminin/DAPI staining of TA muscle in WT and PAI-1 KO mice on day 8 post-injury. Bars represent 100 μm. The box-and-whisker plot shows the number of regenerating (centrally nucleated) and/or restored (peripherally nucleated) skeletal myofibers. More than 10 sections from randomly selected fields per slide were counted for each of three mice per group. ^***^p < 0.001.

To elucidate the role of PAI-1 in inflammation, we examined its expression at the site of inflammation. As previously described [24], CTX, a cytolytic toxin derived from *Naja pallida* that induces transient acute skeletal muscle injury without affecting the vasculature or nerves, was injected into the right TA muscles of C57BL/6 mice (Fig. 1A). This model induces persistent inflammation, followed by synchronized regeneration. The left TA muscle was injected with phosphate-buffered saline (PBS) as a control. Two days after the injection, the mice were euthanized under isoflurane anesthesia, and the TA muscles were harvested. The staining of excised skeletal muscle for cleaved Caspase-3, a key enzyme that is activated during programmed cell death, confirmed that CTX induced apoptotic cell death (Fig. 1B). The collected muscles were finely minced with scissors and treated with collagenase in order to disperse the muscle tissue and isolate nucleated cells enriched with immune cells infiltrating the injury site. RNA was extracted from these nucleated cells, and PAI-1 expression levels were measured by quantitative polymerase chain reaction (qPCR). PAI-1 expression was significantly upregulated in the right TA muscle following CTX treatment (Fig. 1C). These findings demonstrate that PAI-1 expression is strongly induced in skeletal muscle tissue in response to injury-induced inflammation.

### PAI-1 inhibits tissue regeneration

To elucidate the role of PAI-1 in skeletal muscle regeneration following injury, CTX was injected into the right TA muscle of PAI-1 KO mice (Fig. 1D). The mice were euthanized under isoflurane anesthesia 4, 6, and 8 days post injection, and the TA muscles were collected at each time point. The day before euthanasia, Evans blue dye (EBD), which stains damaged or dead cells blue, was intraperitoneally injected to assess cell viability in the tissue. The same procedure was performed on WT mice as controls, and the extent of staining was compared. The results demonstrated that the skeletal muscle of WT mice exhibited robust staining even on day 8. While the PAI-1 KO muscle showed staining comparable to WT muscle on day 4, the signal began to fade by day 6. By day 8, the staining was comparable to that of the PBS-treated muscle (Fig. 1E). This observation suggests that although both WT and PAI-1 KO muscles are similarly damaged by the initial injury, the regeneration process proceeds more rapidly in PAI-1 KO mice.

In order to further investigate the inflammatory response during the early phase of muscle injury, we collected TA muscles from WT and PAI-1 KO mice 4 days after CTX injection and analyzed the expression of inflammatory cytokines using qPCR. The results have revealed that CTX administration markedly upregulated the expression of the pro-inflammatory cytokines, such as TNF-α, IL-1β, IFN-γ, and CCL2, while decreasing the expression of the anti-inflammatory cytokine IL-10. Furthermore, the genetic deletion of PAI-1 resulted in a marked decrease in pro-inflammatory cytokines and an increase in IL-10 expression (Fig. 1F).

To histologically observe the process of skeletal muscle regeneration from injury, tissue sections were prepared and stained with anti-Laminin antibodies (green) and DAPI (blue) to visualize Laminin, a major component of the basal membrane of the skeletal muscle, and nuclei, respectively. The results showed that, whereas skeletal muscle regeneration in the WT mice remained incomplete even on day 8 after injury, PAI-1 KO mice exhibited a well-reconstructed honeycomb-like architecture. Moreover, the proportion of myofibers containing central nuclei (indicative of regenerating muscles) and peripheral nuclei (indicative of mature, functionally restored myofibers) was significantly higher in PAI-1 KO mice, clearly demonstrating that the absence of PAI-1 promotes skeletal muscle regeneration (Fig. 1G). Collectively, these findings indicate that skeletal muscle regeneration is promoted in the absence of PAI-1, suggesting that PAI-1 inhibits skeletal muscle regeneration.

### PAI-1 activity blockade promotes tissue regeneration

**Fig. 2:**
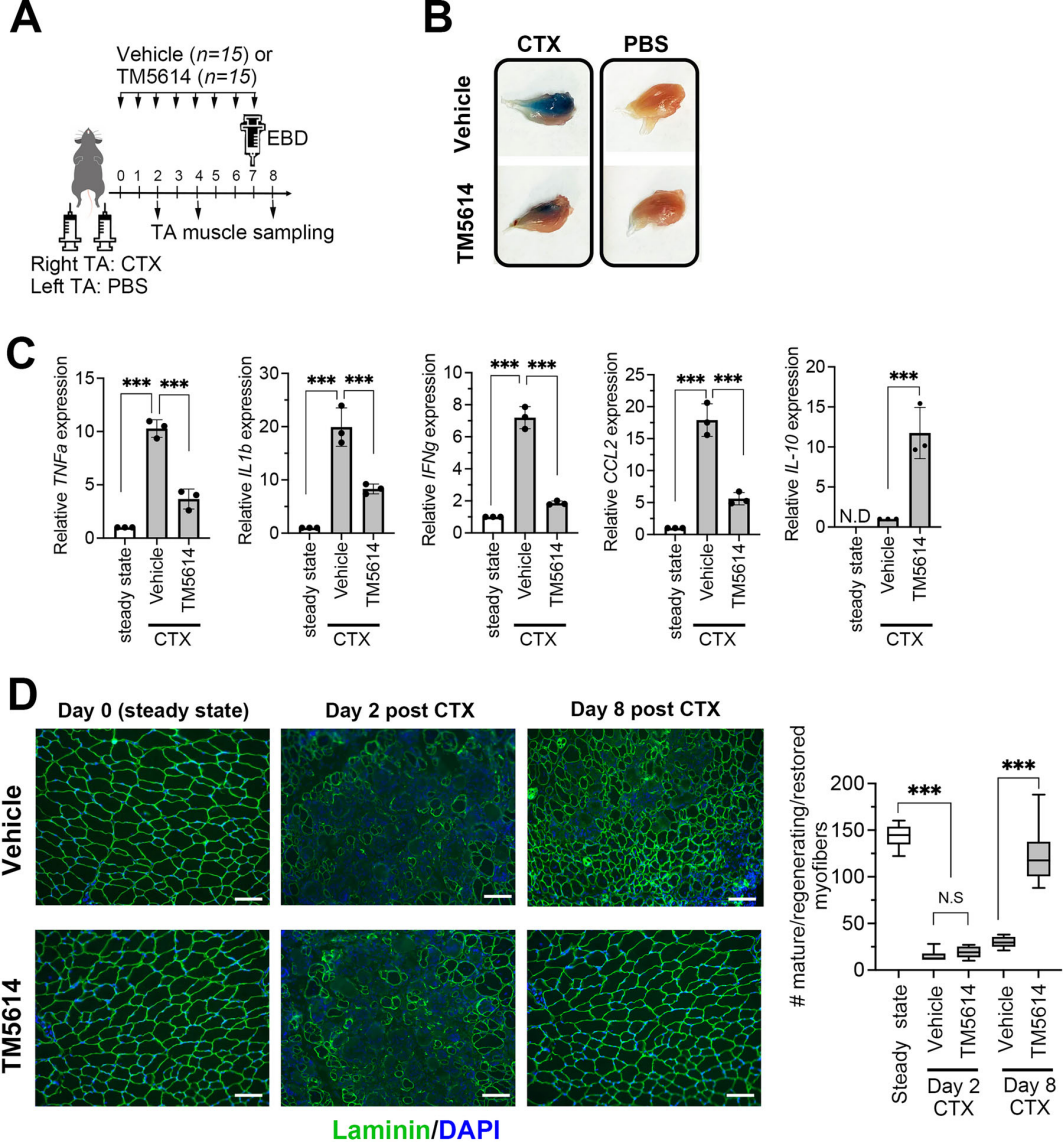
PAI-1 blockade promotes tissue regeneration. **A** Experimental scheme of TM5614 administration after CTX-induced TA muscle injury. Vehicle or 10 mg/kg TM5614 (*n* = 12/group) was administered orally. EBD was injected intraperitoneally into mice 20 h before TA muscle sampling. **B** Representative images of EBD-stained TA muscle in vehicle- and TM5614-treated mice on day 8 post-injury. The original unprocessed images are shown in Supplementary Fig. S3. **C** Pro-inflammatory and anti-inflammatory cytokine mRNA expression in the TA muscles from vehicle- or TM5614-treated mice at day 4 after CTX-induced injury (*n* = 3/group), analyzed by qPCR. Untreated mice were used as steady-state controls. The bars express the results as the mean ± SD of three independent experiments. ^***^p < 0.001. **D** Representative immunohistochemical images of TA muscle showing Laminin and DAPI staining in vehicle- or TM5614-treated mice at days 0, 2, and 8 post-injury. Untreated mice were used as steady-state controls. Bars represent 100 μm. The box-and-whisker plot shows the number of mature (steady-state) or restored skeletal myofibers. More than 10 sections from randomly selected fields per slide were counted for each of three mice per group. ^***^p < 0.001. NS: not significant.

The effect of suppressing PAI-1 activity on enhancing skeletal muscle regeneration was subsequently investigated. C57BL/6 mice were injected with CTX and subsequently administered a PAI-1 inhibitor (TM5614) once daily for five consecutive days (Fig. 2A). Mice in the control group were injected with CTX into the TA muscles and administered oral saline instead of TM5614. On the eighth day after CTX injection, the mice were euthanized under isoflurane anesthesia, and the TA muscles were collected. EBD was injected intraperitoneally the day before euthanasia to assess cell viability. The extent of blue staining was then compared between the groups, revealing that administration of the PAI-1 inhibitor led to a more rapid disappearance of the blue coloration (Fig. 2B). As demonstrated in the PAI-1 KO mice (Fig. 1F, G), both qPCR (Fig. 2C) and histological staining (Fig. 2D) revealed enhanced skeletal muscle regeneration. As demonstrated in the PAI-1 KO mice (Fig. 1F, G), both qPCR (Fig. 2C) and histological staining (Fig. 2D) revealed enhanced skeletal muscle regeneration. Notably, while both WT and PAI-1 KO muscles showed similar levels of damage at day 2, the histological analysis revealed a striking difference in regeneration efficiency by day 8. Repeating the same analyzes in mice treated with a PAI-1 inhibitor yielded similar results, indicating that the pharmacological inhibition of PAI-1 phenocopies the genetic knockout. These findings support the notion that PAI-1 is a critical therapeutic target for inflammatory tissue injury.

### PAI-1 inhibits inflammatory macrophage polarization into suppressive macrophages

**Fig. 3:**
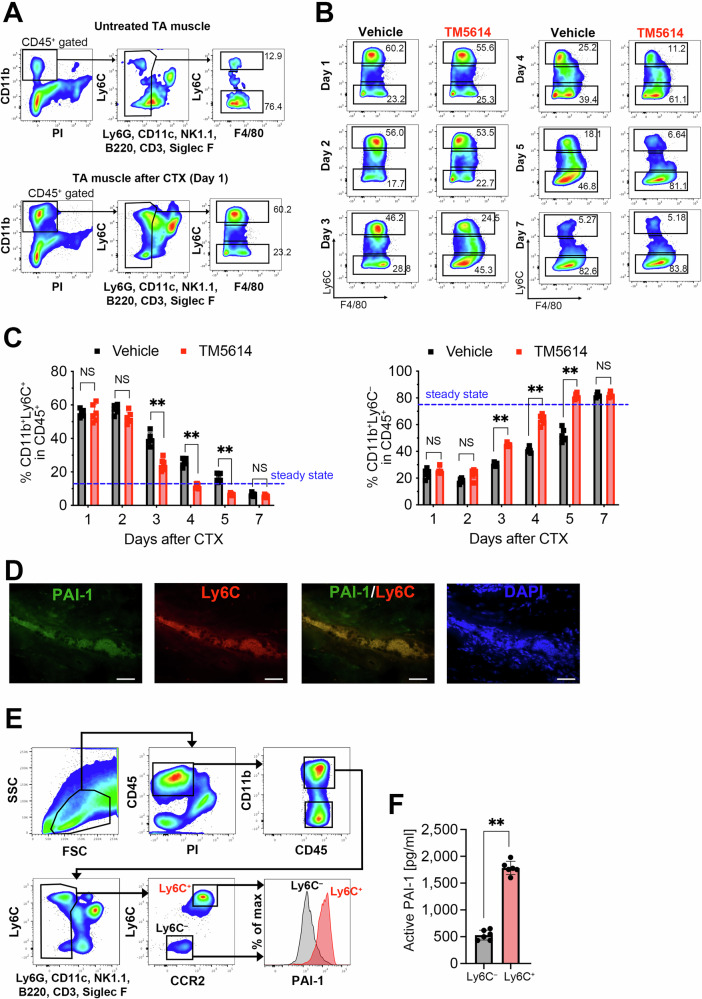
Inflammatory macrophages show high PAI-1 expression. **A** Representative flow cytometric profiles of macrophages in TA muscles sampling from untreated- or CTX-treated muscles on days 1 post-injury. **B** Representative flow cytometric profiles of macrophages in TA muscles sampling from vehicle- or TM5614-treated mice (*n* = 6/group) on days 1, 2, 3, 4, 5, and 7 post-injury. **C** Percentage of CD11b^+^Ly6C^+^ or CD11b^+^Ly6C^−^ macrophages in TA muscles. Data are shown as bar graphs of the results shown in **B**. The bars express the results as the mean ± SD of three independent experiments. The dotted line represents cell percentage at steady state. NS: not significant. **D** Representative immunohistochemical images showing PAI-1 (green), Ly6C (red), and DAPI (blue) staining of macrophages infiltrating CTX-treated TA muscles. Bars represent 100 μm. **E** Representative flow cytometric profiles of PAI-1 and CCR2 in Ly6C^+^ or Ly6C^−^ macrophages in CTX-treated TA muscles. Cells were gated on CD45⁺CD11b⁺CD11c⁻Ly6G⁻NK1.1⁻B220⁻CD3⁻SiglecF⁻PI⁻ to exclude non-macrophage populations and dead cells, and subsequently sorted based on CCR2 and Ly6C expression. **F** Active-form PAI-1 production in macrophages isolated from the CTX-treated TA muscles at day 2 post-injection (*n* = 6), quantified by ELISA. The bars express the results as the mean ± SD. ^**^p < 0.01.

During tissue damage, CCR2^+^Ly6C^+^ monocytes present in the peripheral blood infiltrate the injury site and polarize into Ly6C^+^ inflammatory macrophages, thereby triggering an inflammatory response. This process is accompanied by a marked upregulation of CCL2, a major ligand of CCR2, in damaged skeletal muscle, as shown in Figs. 1F and 2C. Subsequently, Ly6C^−^ suppressive macrophages (also known as repairing macrophages) suppress inflammation, and their balance regulates tissue repair. To investigate how PAI-1 activity inhibition affects macrophage polarization, the temporal dynamics of macrophages present in skeletal muscle were analyzed by flow cytometry. The samples were stained with the pan-leukocyte marker anti-CD45 and lineage markers, including anti-CD3, B220, NK1.1, CD11c, and Ly6G antibodies. PI was also used to stain dead cells. This staining strategy allowed for the exclusion of non-monocyte/macrophage immune cells and dead cells from the performed analysis. Macrophages were identified by staining with the anti-CD11b, F4/80, and Ly6C (Fig. 3A). Flow cytometry was performed by gating on live CD45⁺CD11b⁺ CD3^−^B220^−^NK1.1^−^CD11c^−^Ly6G^−^Siglec F^−^ cells, followed by analysis of Ly6C expression. Percentages represent the proportions of the indicated subsets within the CD45^+^ leukocyte population. The results demonstrated that Ly6C^+^ inflammatory macrophages infiltrated skeletal muscle on day 1 post-injury. However, PAI-1 activity inhibition (*via* TM5614) promoted polarization into suppressive Ly6C^−^ macrophages, with a significant difference observed as early as the third day after muscle injury (Fig. 3B, C). These findings suggested that PAI-1 prolongs inflammation by inhibiting the polarization of inflammatory macrophages into suppressive macrophages.

### Infiltrating CCR2^+^Ly6C^+^ macrophages at the injury site exhibit high PAI-1 expression

To investigate the relationship between infiltrating CCR2⁺Ly6C⁺ macrophages and PAI-1 expression at the injury site, CTX was intramuscularly injected into the TA muscles of C57BL/6 mice. The muscles were harvested on day 2 post injury after euthanasia. Tissue sections were prepared and stained with anti-Ly6C, Ly6G, and PAI-1 antibodies to assess the distribution of Ly6C⁺ macrophages and PAI-1-expressing cells within the injured tissue. This analysis revealed substantial infiltration of Ly6C⁺Ly6G⁻ macrophages (Fig. 3D), indicating their prominent presence at the injury site. Subsequent flow cytometry analysis showed that Ly6C⁺ macrophages at the injury site exhibited elevated PAI-1 expression (Fig. 3E). These Ly6C⁺ cells also expressed CCR2; therefore, in subsequent analyzes, Ly6C⁺ macrophages were defined as CCR2⁺Ly6C⁺ cells. CCR2⁺Ly6C⁺ and CCR2⁻Ly6C⁻ macrophages were isolated and cultured for 2 days, after which active-form of PAI-1 levels in the culture supernatants were measured by ELISA. Consistent with the flow cytometry results, CCR2⁺Ly6C⁺ macrophages secreted significantly higher levels of PAI-1 after CTX treatment (Fig. 3F). These data indicated that CCR2⁺Ly6C⁺ inflammatory macrophages infiltrated the injury site during the early phase of inflammation and robustly secreted PAI-1.

### CCR2^+^Ly6C^+^ inflammatory macrophage-derived PAI-1 exacerbates inflammation

**Fig. 4:**
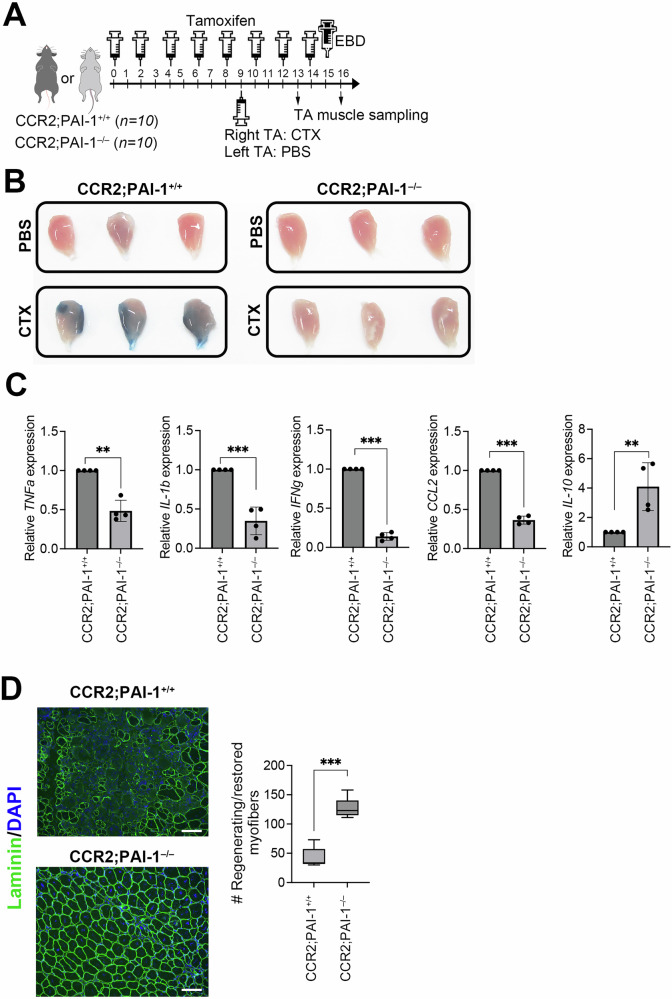
CCR2^+^Ly6C^+^ Inflammatory macrophage-derived PAI-1 exacerbates inflammation. **A** Experimental scheme of CTX-induced TA muscle injury in CCR2;PAI-1^−/−^ mice. **B** Representative images of EBD-stained TA muscle of CCR2;PAI-1^−/−^ mice on day 7 after CTX-induced TA muscle injury following EBD administration (*n* = 6/group). The original unprocessed images are shown in Supplementary Fig. S3. **C** Pro-inflammatory and anti-inflammatory cytokine mRNA expression in TA muscles from CCR2;PAI-1^−/−^ and CCR2;PAI-1^+/+^ mice at day 4 after CTX-induced injury (*n* = 4/group), analyzed by qPCR. The bars express the results as the mean ± SD of three independent experiments. ^**^p < 0.01, ^***^p < 0.001. **D** Representative immunohistochemical images showing Laminin and DAPI staining in CCR2;PAI-1^−/−^ mice on day 7 post-injury. Bars represent 100 μm. The box-and-whisker plot shows the number of regenerating and/or restored skeletal myofibers. More than 10 sections from randomly selected fields per slide were counted for each of three mice per group. ^*^p < 0.05, ^***^p < 0.001.

To elucidate the role of PAI-1-high-producing CCR2^+^Ly6C^+^ macrophages in inflammation and regeneration, we generated a conditional KO mouse lacking PAI-1 specifically for CCR2^+^cells: PAI-1-floxed mouse (PAI-1-floxed mouse generation is shown in Supplementary Fig. S1A, B) with tamoxifen-inducible expression of Cre recombinase under the control of the CCR2 promoter [CCR2-CreER-GFP^+^;PAI-1^flox/flox^ mouse, hereafter referred to as CCR2;PAI-1^−/−^ mouse]. CCR2-CreER-GFP^−^;PAI-1^flox/flox^ mouse (CCR2;PAI-1^+/+^) served as a control group. Tamoxifen was administered intraperitoneally five times at 2-day intervals. This mouse line expresses GFP under the control of the CCR2 promoter; therefore, GFP⁺ cells represent CCR2⁺ cells. To confirm the specificity of the PAI-1 deletion, we isolated GFP⁺ cells after tamoxifen administration and analyzed the expression of the PAI-1 gene and protein. Supplementary Fig. S1C, D show that PAI-1 was efficiently deleted from these cells. Two days after the last dose, CTX and PBS were injected into the right and left TA muscles, respectively. Tamoxifen administration was continued after CTX injection because the CCR2^+^ cells were continuously replaced. On the eighth day after injection, the mice were euthanized and the TA muscles were collected (Fig. 4A). The regeneration process following skeletal muscle injury was compared between the two groups 7 days after CTX injection using EBD administration (Fig. 4B), qPCR analysis of inflammatory cytokines (Fig. 4C), and fluorescent immunostaining for Laminin and nuclei in skeletal muscle sections (Fig. 4D). The results showed that the skeletal muscles of CCR2;PAI-1⁺/⁺ mice remained strongly stained with EBD even on day 8, whereas those of CCR2;PAI-1⁻/⁻ mice were similar to PBS-injected controls (Fig. 4B). Furthermore, the qPCR analysis of TA muscles collected on day 4 revealed that the expression of pro-inflammatory cytokines, including TNF-α, was significantly decreased in CCR2;PAI-1⁻/⁻ mice when compared to CCR2;PAI-1⁺/⁺ controls, while anti-inflammatory cytokine, IL-10, was upregulated. Immunostaining demonstrated rapid reconstruction of the skeletal muscle honeycomb-like structure in CCR2;PAI-1⁻/⁻ mice (Fig. 4C). In addition, the numbers of central and peripheral nuclei were found to be significantly increased in CCR2;PAI-1⁻/⁻ mice (Fig. 4D). These results phenocopied those obtained in global PAI-1 KO mice (Fig. 1), thus suggesting that PAI-1 expression in CCR2⁺ cells is a major factor responsible for delayed muscle regeneration.

### CCR2^+^Ly6C^+^inflammatory macrophage-derived PAI-1, but not environmental PAI-1, exacerbates inflammation

**Fig. 5:**
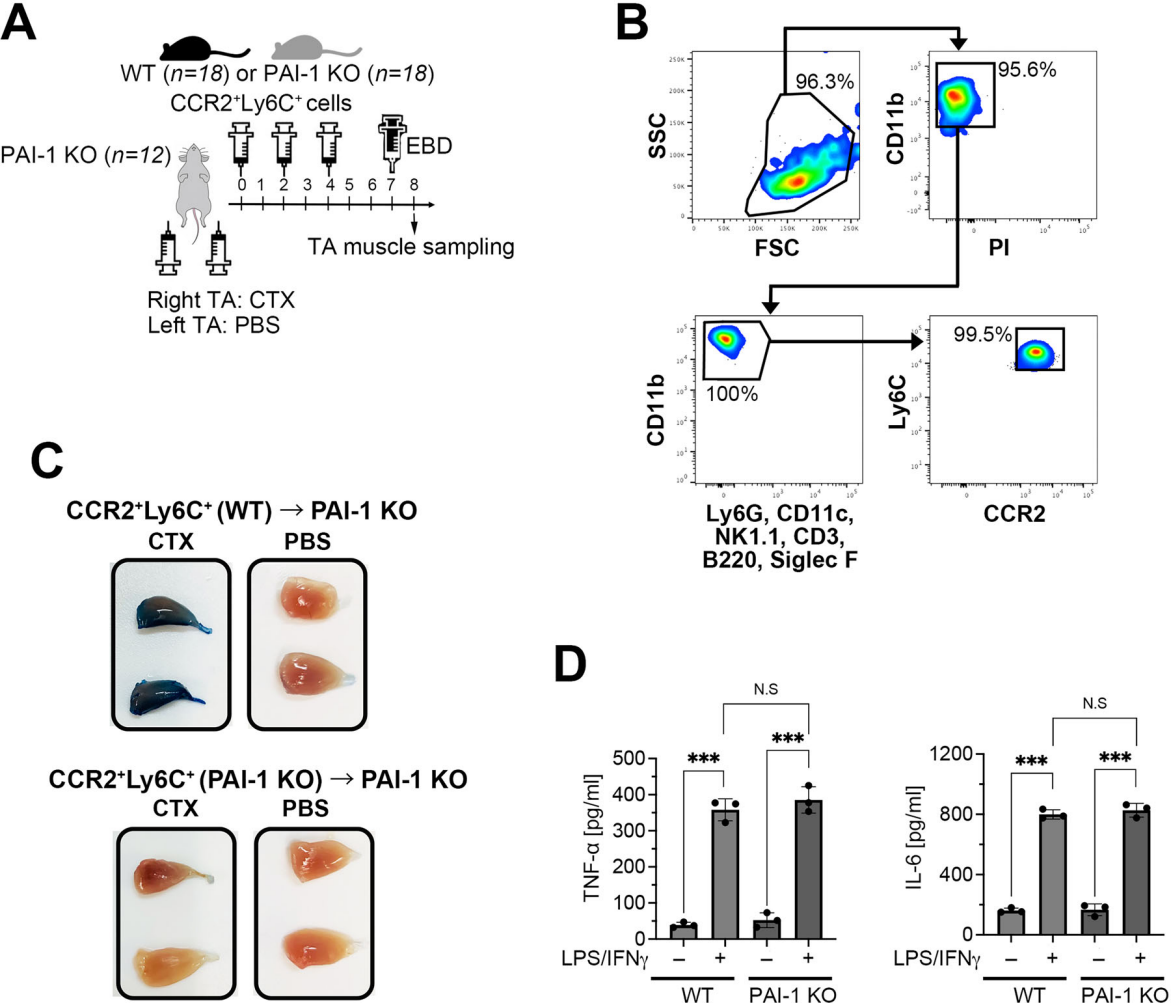
Inflammatory macrophage-derived PAI-1, but not environmental PAI-1, exacerbates inflammation. **A** Experimental scheme of adoptive transplantation of CCR2^+^Ly6C^+^ BM-derived macrophages from WT or PAI-1 KO mice (*n* = 6/group) into CTX-treated PAI-1 KO recipient mice. **B** Representative flow cytometric profiles of CCR2^+^Ly6C^+^ BM-derived macrophages for adoptive transplantation. **C** Representative images of EBD-stained TA muscle from recipient mice on day 8 after CTX-induced TA muscle injury following EBD administration. **D** TNF-α and IL-6 production in the CCR2^+^Ly6C^+^ macrophages isolated from the WT and PAI-1 KO mice at day 2 post-LPS and IFN-γ stimulation (*n* = 3), quantified by ELISA. The bars express the results as the mean ± SD. ^**^p < 0.01. NS: not significant.

To clarify whether the pro-inflammatory role of PAI-1 originates from CCR2⁺Ly6C⁺ cells rather than the surrounding environment, we conducted an adoptive transfer experiment using PAI-1 KO mice—which systemically lack PAI-1—as recipients, and CCR2⁺Ly6C⁺ cells derived from either WT or PAI-1 KO donors (Fig. 5A). Furthermore, because CCR2 is expressed in several immune cell types beyond macrophages, this experiment was also designed to specifically determine whether the observed inflammatory phenotype could be attributed to PAI-1 produced by CCR2⁺Ly6C⁺ macrophages. Most of the sorted cells (98%) were CD11b^+^CCR2^+^Ly6C^+^ (Fig. 5B) and intravenously transplanted three times every alternate day into PAI-1 KO mice that received CTX injections. On the eighth day after CTX injection, the mice were euthanized under isoflurane anesthesia, and the TA muscles were collected. EBD was injected intraperitoneally the day before euthanasia. The results showed prolonged inflammation and delayed regeneration only after transplanting PAI-1-high-producing CCR2^+^Ly6C^+^ macrophages from WT mice (Fig. 5C).

To exclude the possibility that these differences arose from an intrinsic defect in macrophage inflammatory polarization caused by PAI-1 deficiency, we conducted supplementary in vitro experiments using BM–derived CCR2^+^Ly6C^+^ macrophages from WT and PAI-1 KO mice. The cells were stimulated with IFN-γ and LPS for 2 days, and cytokine production was assessed. No significant differences were observed in the levels of the pro-inflammatory cytokines, such as TNF-α and IL-6, following LPS/INF-γ stimulation (Fig. 5D). These results suggest that PAI-1 deficiency does not markedly affect the intrinsic pro-inflammatory potential of macrophages and that other mechanisms are likely involved in the observed phenotype.

These results indicated that although PAI-1 is abundant in body fluids, its presence or absence of PAI-1 in the surrounding environment does not affect CCR2^+^Ly6C^+^ macrophage-induced inflammation progression. However, PAI-1 expression by macrophages prolonged the inflammatory response and delayed the regenerative process. This finding provides direct evidence that CCR2^+^Ly6C^+^ macrophages producing high levels of PAI-1 play a pivotal role in exacerbating inflammation.

### PAI-1 suppresses the efferocytosis activity of macrophages

**Fig. 6:**
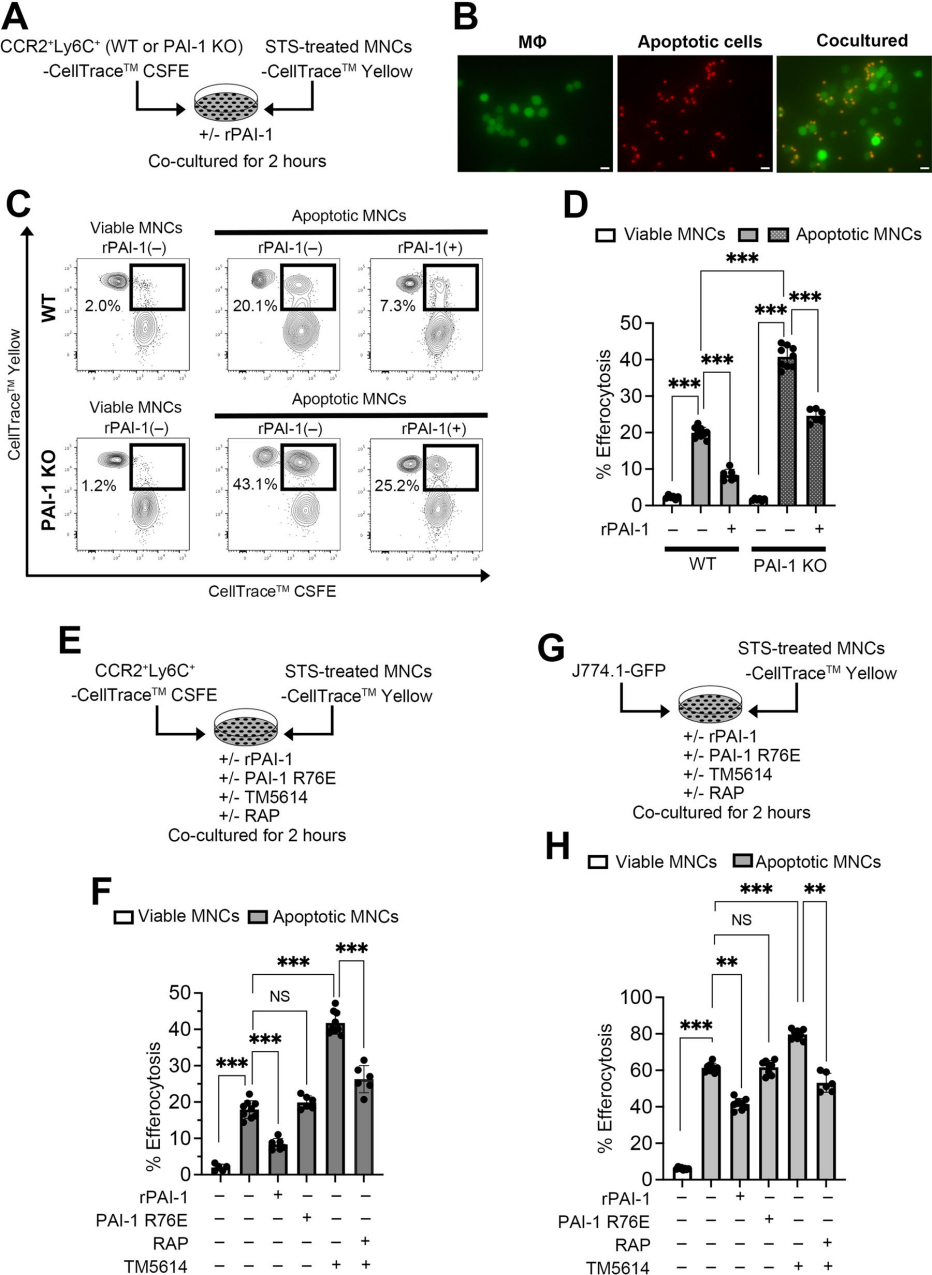
PAI-1 suppresses the efferocytosis activity of macrophages. **A** Experimental scheme of the in vitro efferocytosis assay corresponding to **B**–**D**. **B** Representative fluorescence images of macrophages (green) and apoptotic cells (red). Yellow signals indicate the macrophages that have engulfed apoptotic cells. Bars represent 20 μm. **C** Representative flow cytometric profiles of the in vitro efferocytosis assay. **D** Percentage of CCR2^+^Ly6C^+^ BM-derived macrophages from CTX-treated WT or PAI-1 KO mice (*n* = 6/group) that phagocytosed dead cells. **E** Experimental scheme of the in vitro efferocytosis assay corresponding to **F**. **F** Percentage of CCR2^+^Ly6C^+^ BM-derived macrophages from CTX-treated WT mice that phagocytosed dead cells (*n* = 6/group). **G** Experimental scheme of the in vitro efferocytosis assay corresponding to **H**. **H** Percentage of J774.1-GFP cells that phagocytosed dead cells (*n* = 6/group). The bars express the results as the mean ± SD of three independent experiments. ^**^p < 0.01, ^****^p < 0.0001. NS: not significant.

To elucidate the mechanism through which PAI-1 produced by CCR2^+^Ly6C^+^ macrophages exacerbates inflammation, we conducted an in vitro analysis focusing on efferocytosis (Fig. 6A). CTX was injected into the right TA muscle of WT mice or PAI-1 KO mice. Three days later, the mice were euthanized, and the BM was collected. CCR2^+^Ly6C^+^ macrophages were isolated from the BM cells and stained with green fluorescence dye. To prepare dead cells, untreated C57BL/6 mice were euthanized, and their spleens were harvested and dissociated to isolate mononuclear cells (MNCs), which were cultured overnight with staurosporine (STS), a prototypical ATP-competitive kinase inhibitor, to induce apoptotic cell death (Supplementary Fig. S4A). The dead cells were then stained with red fluorescence dye and co-cultured with green fluorescence dye-labeled CCR2^+^Ly6C^+^ macrophages. After 2 h, the contact and uptake of dead cells by macrophages resulted in yellow fluorescence, clearly indicating efferocytosis (Fig. 6B). This yellow signal, formed by the internalization of red dead cells into green macrophages, was confirmed by fluorescence microscopy and quantified as double-positive cells by flow cytometry (Fig. 6C). Taken together, these serve as reliable indicators of efferocytic activity.

To elucidate the role of PAI-1 in efferocytosis, CCR2^+^Ly6C^+^ macrophages isolated from WT and PAI-1 KO mice were co-cultured with STS-treated dead cells. After 2 h, macrophages were collected, and the extent of phagocytosis was analyzed by flow cytometry (Fig. 6C). The results demonstrated that PAI-1 deficiency significantly enhanced the phagocytic capacity of macrophages (Fig. 6D). Conversely, treatment with rPAI-1 reduced the proportion of efferocytic macrophages (Fig. 6D). Importantly, even in the absence of exogenous rPAI-1, a significant difference in phagocytic activity was observed between WT and PAI-1 KO macrophages (Fig. 6D). This was likely because of the spontaneous production of PAI-1 by the CCR2^+^Ly6C^+^ macrophages under steady-state culture conditions, as shown in Supplementary Fig. S4B. These results demonstrate that PAI-1 suppresses the efferocytic activity of macrophages. Of particular importance is the finding that PAI-1 produced by macrophages controls efferocytosis by both autocrine and paracrine mechanisms under physiological conditions.

### Efferocytosis suppression by PAI-1 requires its binding to LRP-1

When cells undergo cell death, they begin to express CRT, which is recognized by LRP-1 on macrophages. This recognition transmits an “eat me” signal and leads to the phagocytic removal of dying cells. We confirmed that CRT was expressed on the surface of CTX-treated TA muscle cells (Supplementary Fig. S4C) and STS-treated cells (Supplementary Fig. S4D), whereas LRP-1 was expressed on the surface of macrophages, including CCR2⁺Ly6C⁺ primary macrophages and J774.1 macrophage cell lines (Supplementary Fig. S4E). As LRP-1 also serves as a receptor for PAI-1, we hypothesized that PAI-1 may competitively inhibit CRT recognition by macrophages. To test this, red-labeled dead cells were co-cultured with green-labeled CCR2⁺Ly6C⁺ macrophages (Fig. 6E) or GFP-expressing J774.1 cells (J774.1-GFP cells) (Fig. 6G), in the presence or absence of PAI-1 R76E, a mutant form of PAI-1 deficient in LRP-1 binding. No inhibitory effect on efferocytosis was observed with the LRP-1-binding-deficient mutant (Fig. 6F, H), indicating that suppression of efferocytosis by PAI-1 is dependent on its ability to bind LRP-1.

### PAI-1 competitively inhibits CRT binding to LRP-1

**Fig. 7:**
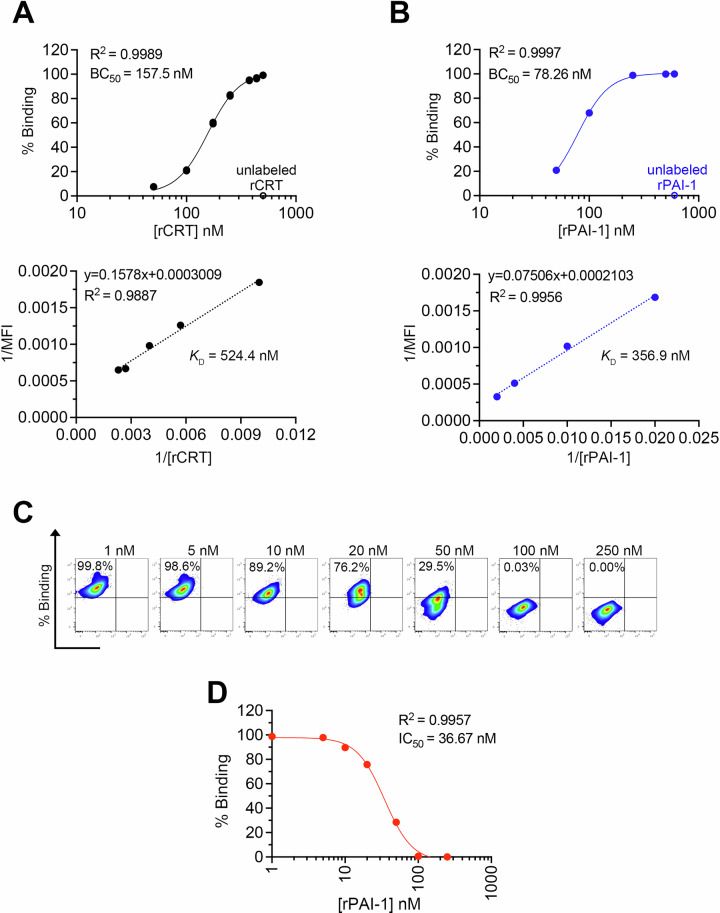
PAI-1 inhibits CRT binding to LRP-1. **A** Correlation between the concentration of NHS-labeled rCRT and the percentage of its binding to LRP1 on J774.1 cells. **B** Correlation between NHS-labeled rPAI-1 concentration and the percentage of its binding to LRP1 on J774.1 cells. **C** Representative flow cytometric profiles of the competitive binding assay. **D** Correlation of rPAI-1 concentration with the percentage of NHS-labeled rCRT binding to LRP1 on J774.1 cells. *R*^*2*^ value is calculated using nonlinear regression analysis (4 parameter logistic regression).

To demonstrate that PAI-1 inhibited the interaction between CRT and LRP-1, a competitive binding assay was performed using recombinant proteins. Initially, rCRT or rPAI-1 was labeled with NHS ester−fluorescein dye. Each NHS-labeled protein was serially diluted and added to LRP-1-expressing J774.1 cells. The extent of binding was analyzed using flow cytometry, which revealed ligand concentration-dependent binding. The 50% binding concentration (BC_50_) for rCRT (Fig. 7A) and rPAI-1 (Fig. 7B) were 157.5 and 78.26 nM (*R*^2^ = 0.9989 and 0.9997), respectively. When pre-incubation was performed with unlabeled ligands, NHS-labeled ligand binding was no longer observed (Fig. 7A, B), indicating that the binding was specific. These results indicated that PAI-1 saturates LRP-1 at a lower concentration than CRT. The dissociation constant (*K*_D_) for rCRT (Fig. 7A) and rPAI-1 (Fig. 7B) was 524.7 and 352.4 nM (*R*^2^ = 0.9887 and 0.9956), respectively, indicating that PAI-1 has a higher affinity for LRP1 than CRT. The inhibitory effect of rPAI-1 on the binding of rCRT to LRP-1 was examined by adding serially diluted rPAI-1 to 500 nM rCRT. The results demonstrated that PAI-1 significantly inhibited CRT binding to LRP-1 (Fig. 7C) with a median inhibition concentration (IC_50_) of 36.67 nM (*R*^2^ = 0.9957) (Fig. 7C). These findings unequivocally demonstrate that PAI-1 exhibits a competitive binding affinity for LRP-1, thereby impeding efferocytosis.

### PAI-1 activity blockade promotes macrophage efferocytosis

The results of this study suggest that PAI-1 activity suppression likely enhances macrophage efferocytosis. During co-culture under the same procedures as in Fig. 6E, G, TM5614 was added. After a 2-h incubation period, the macrophages were collected, and phagocytosis extent was analyzed by flow cytometry. The results demonstrated that the presence of the PAI-1 inhibitor increased the phagocytosed macrophage proportion (Fig. 6F, H, and Supplementary Fig. S5). This finding suggests that the PAI-1 inhibitor enhances the efferocytotic activity of the macrophages. In the same experimental system, specific LRP-1 inhibition with RAP, an LRP-1 antagonist, counteracted the enhancement of efferocytosis by TM5614 (Fig. 6F, H, and Supplementary Fig. S5), indicating that TM5614 specifically inhibited the PAI-1–LRP-1-mediated reaction.

**Fig. 8:**
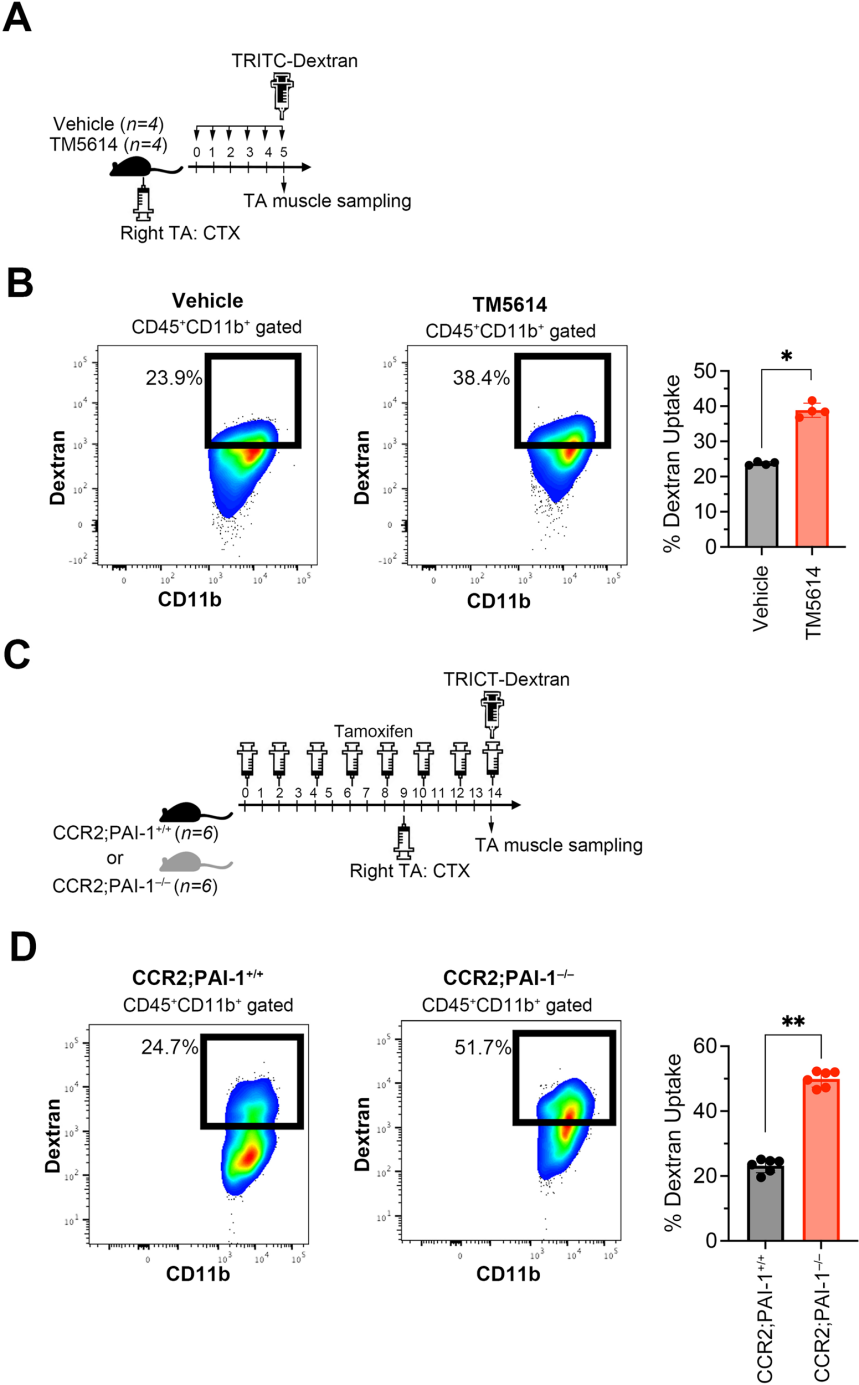
PAI-1 inhibition promotes macrophage efferocytosis in vivo. **A** Experimental schema of TRITC–Dextran uptake in CCR2^+^Ly6C^+^ macrophages infiltrating into TA muscles of vehicle- or TM5614-administered CTX-treated mice (*n* = 4/group). Mice were injected intraperitoneally with TRITC–Dextran 2000 kDa 3 h before TA muscle sampling. **B** Representative flow cytometric profiles and percentage of TRITC–Dextran 2000 kDa uptake in vehicle- or TM5614-treated macrophages. **C** Experimental schema of TRITC–Dextran uptake in macrophages infiltrating into TA muscles of CTX-treated CCR2;PAI-1^+/+^ or CCR2;PAI-1^−/−^ mice (*n* = 6/group). Mice were injected intraperitoneally with TRITC–Dextran 2000 kDa 3 h before TA muscle sampling. **D** Representative flow cytometric profiles and percentage of TRITC–Dextran 2000 kDa uptake in macrophages from CCR2;PAI-1^+/+^ or CCR2;PAI-1^−/−^ mice. The bars are expressed as the mean ± SD of three independent experiments. ^*^p < 0.05, ^**^p < 0.01.

To verify that the efferocytosis-promoting effect of the PAI-1 inhibitor also occurred in vivo, CTX was injected into the right TA muscles of C57BL/6 mice (Fig. 8A). On the same day, the mice were orally administered a PAI-1 inhibitor for three consecutive days, while the control group received saline. On the day following the final oral administration, TRITC-labeled dextran was injected intraperitoneally. Three hours later, the mice were euthanized, and the right TA muscles were collected. The muscles were digested with collagenase, and mononucleated cells were isolated. Dextran uptake was then analyzed by flow cytometry. Although dextran is not a direct marker of dead cell engulfment, it serves as an indirect indicator of macrophage phagocytic activity, which we interpret as a surrogate measure of efferocytic capacity under inflammatory conditions. The results showed that PAI-1 inhibitor treatment increased dextran uptake by macrophages (Fig. 8B). Finally, to confirm that PAI-1 produced by CCR2⁺Ly6C⁺ macrophages plays a key role in regulating efferocytosis in vivo, we used CCR2;PAI-1⁻/⁻ mice and assessed TRITC-dextran uptake following CTX administration (Fig. 8C). As expected, selective PAI-1 depletion in CCR2⁺Ly6C⁺ macrophages enhanced dextran uptake (Fig. 8D), indicating that macrophage-derived PAI-1 impairs efferocytosis in vivo, thereby exacerbating inflammation and hindering tissue regeneration.

## Discussion

PAI-1 is a multifunctional protein that not only regulates the fibrinolytic system but also controls various biological phenomena [17]. However, many aspects of its role as a malignant factor in numerous diseases remain unclear. In this study, we focused on the inflammatory response driven primarily by inflammatory macrophages, which are a major cause of many diseases, and successfully elucidated the functional relationship between PAI-1 and prolonged macrophage-mediated inflammation. Notably, despite the PAI-1 abundance in body fluids, we have successfully demonstrated that elevated PAI-1 production by CCR2^+^Ly6C^+^ inflammatory macrophages at the local inflammation site plays a critical role in disease pathogenesis. Our study has identified a novel role for PAI-1 in inflammation progression and resolution; PAI-1 suppresses macrophage efferocytosis through the competitive inhibition of “eat me” signaling, thereby impeding damaged or dead cell clearance and acting as a key factor in inflammation prolongation. Furthermore, TM5614, a PAI-1 inhibitor developed from the lead compound TM5275 [25–31], significantly improves inflammation-associated tissue damage and promotes tissue regeneration. These findings indicate that macrophages with high PAI-1 production play a central role in inflammation pathogenesis and establish PAI-1 as a therapeutic target for inflammatory conditions.

PAI-1 primarily regulates blood coagulation and fibrosis through the fibrinolytic system [17]. In this study, we identified a new mechanism by which PAI-1 acts directly on cells to regulate efferocytosis. Resolution of inflammation necessitates the efficient clearance of damaged or dead cells generated during the inflammatory response, thereby creating an environment conducive to tissue regeneration [32]. Efferocytosis is critical for maintaining tissue homeostasis [8, 9]. Notably, in chronic inflammatory diseases, impaired efferocytosis accumulates damaged and dead cells, along with the accumulation of damage-associated molecular patterns, pathogen-associated molecular patterns, and senescence-associated secretory phenotypes, which are dispersed from their remnants and contribute to persistent inflammation [33–36]. In this study, we discovered that PAI-1 hinders the “eat me” signals, thereby suppressing macrophage efferocytosis. This inhibition prevents the clearance of dead cells from damaged tissue during inflammation, potentially leading to persistent chronic inflammation. Our findings reveal a novel role for the inflammation-associated protein PAI-1 in exacerbating inflammation. Although previous studies have shown that elevated PAI-1 expression in dying cells impairs macrophage-mediated efferocytosis [37], our study provides the first evidence of a regulator *via* autocrine and/or paracrine mechanisms. Importantly, our experimental data demonstrate that macrophage-specific deletion of PAI-1 significantly enhances efferocytosis, indicating that macrophage-derived PAI-1 in local inflammation plays a more dominant role in suppressing this process than PAI-1 originating from dead cells or extracellular fluids. This may be attributed to the large number of inflammatory macrophages accumulating at sites of inflammation, the substantial amount of PAI-1 they secrete, and the increased conversion of PAI-1 from its inactive to active form—all of which may more strongly contribute to disease progression.

Of note, this is the first demonstration that PAI-1 competitively inhibits the binding of CRT to LRP-1. PAI-1 demonstrated more than twice the affinity for LRP1 than CRT, suggesting that PAI-1 is a significant inhibitor of regeneration at the inflammation site. PAI-1 is a ligand for LRP-1; however, when it forms a complex with the urokinase-type plasminogen activator (uPA), it has 100 times the binding capacity of its free form [38]. Therefore, the inhibitory effect should be even stronger under physiological conditions. These results suggest that no matter how many dead cells present the “eat me” signal, the presence of PAI-1 creates a situation in which dead cells are not eliminated indefinitely, and tissue repair is delayed. These findings are critical for understanding the biological phenomena underlying inflammation. Our study focused on the competitive inhibition between CRT and PAI-1 for LRP-1 binding. However, efferocytosis is also regulated by “don’t eat me” signals, such as CD47. Future studies may reveal how PAI-1 interacts with other regulatory systems to fine-tune phagocytic responses during inflammation.

Macrophages are deeply involved in several inflammatory diseases. Therefore, although our current study focuses on skeletal muscle injury, the involvement of macrophage-derived PAI-1 in prolonging inflammation may represent a generalizable mechanism applicable to various chronic inflammatory diseases, such as colitis, arthritis, or atherosclerosis. This discovery contributes to the understanding of the mechanisms underlying disease pathogenesis and the development of novel therapeutic strategies. Moreover, accumulating evidence indicates that macrophage-mediated phagocytosis also plays a pivotal role in cancer progression [39]. We have previously reported that PAI-1 negatively regulates T cell-mediated anti-tumor immunity and enhances cancer malignancy [27]. The current study extends these findings by suggesting that PAI-1 also impairs the clearance of cancer cells by macrophages through efferocytosis inhibition. Collectively, these findings suggest that PAI-1 modulates both innate and adaptive immunity, positioning it as a key regulator of immune homeostasis in diverse pathologies, including chronic inflammation and cancer. This dual role further underscores the therapeutic potential of targeting PAI-1 across disease contexts.

In addition to its regulatory function in phagocytic activity, PAI-1 also influences macrophage motility and polarization [40, 41]. Within the context of cancer, PAI-1 exacerbates malignancy by recruiting macrophages to tumor sites and polarizing them into M2-type macrophages, which suppress tumor immunity [42]. However, the results of this study suggest that at inflammatory sites, PAI-1 may instead act to inhibit macrophage polarization into suppressive phenotypes. Although inflammation and cancer have many commonalities, the mechanisms underlying PAI-1 actions may differ owing to the variations in PAI-1-inducing cytokines, such as TNF-α or TGF-β, or differences in the time scale of disease progression. In any case, excess PAI-1 production appears to direct macrophage functions toward exacerbating inflammation or cancer malignancy.

Several studies have reported that PAI-1 is highly expressed in patients with inflammatory conditions and has been implicated in tissue fibrosis [17, 43]. Additionally, the symptoms of inflammatory disease are alleviated in PAI-1-deficient mice [44]. These studies suggest that PAI-1 promotes inflammation through the fibrinolytic system; however, they did not focus on inflammatory cell function regulation, as examined in this study. Although these findings indicate that PAI-1 is a promising therapeutic target in inflammatory diseases, no previous studies have demonstrated the therapeutic effects of PAI-1 inhibitors. In this study, we showed that a PAI-1 inhibitor significantly improved symptoms, including tissue reconstruction promotion, in a skeletal muscle injury inflammation model. These results unequivocally establish PAI-1 as a pivotal exacerbating factor in inflammation pathogenesis and directly demonstrate that PAI-1 is a therapeutic target in inflammatory pathogenesis. We have previously reported that a PAI-1 inhibitor regulates not only the fibrinolytic system but also stem cell motility and immune checkpoint molecule expression [27, 30, 45–47]. In this study, we demonstrated its unique function as a promoter of efferocytosis. This underscores the fact that TM5614 exerts its effects through a mechanism of action that is distinct from that of conventional anti-inflammatory therapies, such as steroid drugs and cytokine inhibitors, making it a particularly noteworthy development. A notable advantage of TM5614 is its oral administration, which makes it a promising new option for anti-inflammatory treatment, with the potential to reduce side effects. It is a novel drug whose efficacy and safety have been demonstrated in multiple clinical trials, primarily for several cancers [29, 31, 48–52]; its effectiveness is anticipated in the treatment of inflammatory diseases.

## Materials and methods

### Reagents and resources

The list of reagents and resources used in this study, including antibodies, is presented in Supplementary Table S1.

### PAI-1 inhibitor

TM5614, a small molecule inhibitor of PAI-1, selectively blocks the interaction between PAI-1 and its target serine proteases, thereby enhancing plasmin generation. Its high specificity for PAI-1 over other serpins has been demonstrated previously [25]. A tPA-dependent hydrolysis assay confirmed that TM5614 functioned as an effective PAI-1 inhibitor, with a significant reduction in regard to PAI-1 activity observed at IC₅₀ of less than 6.95 μM.

### Cell line

J774.1, a murine macrophage-like cell line, was obtained from the RIKEN Cell Bank (RRID: CVCL_4770; Ibaraki, Japan) and transduced with a retrovirus carrying an EGFP vector in order to generate J774.1-GFP cells. The cells were cultured in RPMI-1640 medium (FujiFilm Wako Pure Chemical, Osaka, Japan) supplemented with 10% heat-inactivated fetal bovine serum (FBS; Biosera, Cholet, France), 2 mM L-glutamine (FujiFilm Wako Pure Chemical), and antibiotics (100 U penicillin/mL and 100 μg streptomycin/mL; Thermo Fisher Scientific, Waltham, MA, USA) at 37 °C in a humidified atmosphere containing 5% CO_2_. The cell line has been confirmed to be mycoplasma-free.

### CCR2^+^Ly6C^+^ macrophages

Murine bone marrow (BM) was flushed with consisting of PBS, 2 mM EDTA, and 0.5% bovine serum albumin (BSA; FujiFilm Wako Pure Chemical). Subsequently, the cell suspensions were passed through a 40 μm nylon cell strainer (Corning, Corning, NY, USA) and incubated for 5 min with ACK (ammonium-chloride-potassium) lysis buffer to lyse the red blood cells. To enrich CCR2^+^Ly6C^+^ macrophages, BM MNCs were suspended in PEB buffer and treated with the Fcγ receptor-blocking reagent. Negative selection was performed using a biotin-conjugated antibody cocktail targeting T cells, B cells, NK cells, dendritic cells, erythroid cells, and granulocytes, followed by separation using MACS columns (Mintenyi Biotec, Bergisch Gladbach, Germany).

### Laboratory animal care and use

Twelve- to twenty-week-old male C57BL/6J mice were obtained from CLEA Japan, Inc. (Tokyo, Japan). PAI-1-knockout (PAI-1 KO) mice (B6.129S2-Serpine1^tm1Mlg^/J, RRID: IMSR_JAX:002507) and CCR2-CreER-GFP mice [C57BL/6-Ccr2^em1(icre/ERT2)Peng^/J, RRID: IMSR_JAX:035229] were purchased from the Jackson Laboratory (Bar Harbor, ME, USA). CCR2-CreER-GFP mice were mated with PAI-1-floxed mice to obtain the CCR2-CreER-GFP(+);PAI-1^flox/flox^ offspring. Littermates, specifically CCR2-CreER-GFP(−) and PAI-1^flox/flox^ mice, served as the control group. In these mice, 60 mg/kg tamoxifen or vehicle (corn oil) was administered every other day, five times before use, and continued after the experiment began because the CCR2^+^ cells were continuously replaced. The generation of PAI-1-floxed and skeletal muscle injury model mice is described below. The mice were housed under controlled humidity (50 ± 10%) and temperature (23 ± 2 °C), with a standard 12 h light/dark cycle and provided *ad libitum* access to sterile water and a pellet diet in a barrier and specific-pathogen-free environment. All of the mice were euthanized *via* cervical dislocation under full anesthesia induced by inhaled isoflurane (2.5% in air during anesthesia) by trained personnel in accordance with the American Veterinary Medical Association (AVMA) Guidelines for the Euthanasia of Animals (2020 Edition).

### PAI-1-floxed mouse generation

CRISPR guide RNAs targeting introns 3 and 5 of *Serpine1* (PAI-1) were designed using CRISPOR (https://crispor.gi.ucsc.edu/) [53]. The annealed crRNAs (PAI1-Cr3: TGCCCCCTAAGCTATAGTCT, or PAI1-Cr4: CTTGCCCCCATAGCCAAAGC) and tracrRNA were mixed with Cas9 protein and ssODNs so that the final concentrations of the components in electroporation solution were 15 μM (for each crRNA/tracrRNA), 1 mg/mL (for Cas9 protein), 1 μg/μL (for each ssODN; PAI1_5′_ssODN: ATAAATAAACAAAACCAAAATCACCATGAAAGGGTACCACTGTCAGTAAGAACAACAGGAGCTGTAACACAGGTGTACACGGGCCTCCAAGAataacttcgtatagcatacattatacgaagttatCTATAGCTTAGGGGGCAAGAGAAGAGGTCAAATGA, and PAI1_3′_ssODN: GTAAGAGTCAACCAAAGTGAATCATAGTGGCACAAGCCTGTAACCCTAGTGTTAGACAGGCTGAGACAGAATAGTGAGTTCAAGACCAGCTataacttcgtatagcatacattatacgaagttatTTGGCTATGGGGGCAAGATTGCTGGCTTACTTTGTA). Improved genome editing *via* oviductal nucleic acid delivery (*i*-GONAD) was performed using anesthetized C57BL/6 females on day 0.7 of pregnancy, as described previously [54, 55]. The ovary/oviduct was exposed after an incision was made on the dorsal skin. Approximately 1.0–1.5 μL electroporation solution (pre-warmed at 37 °C for 10 min) was injected into the oviduct lumen from upstream of the ampulla. Electroporation was performed using a square-wave pulse generator (CUY21EDIT II; BEX). The electroporation parameters were as follows: square (mA), (+/−), Pd V: 80 V, Pd A: 100 mA, Pd on: 5.00 ms, Pd off: 50 ms, Pd N: 3, Decay: 10%, Decay Type: Log. Mouse genomic DNA was extracted from ear samples of newborns and then subjected to PCR-based genotyping using primers (M1233: GTGTACACGGGCCTCCAAGAataacttcgtatagc, M1234: AATCTTGCCCCCATAGCCAAataacttcgtataat, M1238: CCCCTTGGCCAGTAAGTCAC, M1239: CCAACATCTTGGATGCTGAA) to identify the floxed allele. Targeted loxP insertions were assessed by sequencing using the Genetic Analyser 3500XL (Thermo Fisher Scientific). Additional information regarding the mice is provided in Supplementary Fig. S1. The original unprocessed images from the PCR-based analysis are shown in Supplementary Fig. S2.

### Cardiotoxin-induced skeletal muscle injury

Skeletal muscle injury was induced by injecting cardiotoxin (CTX) as described previously [24]. Briefly, the mice were anesthetized with isoflurane, and 50 μL CTX (12.5 μM in PBS; Latoxan, Valence, France) was injected in the right tibialis anterior (TA) muscle using 27G needle insulin syringe. The left TA muscle was injected with PBS as a control. The TA muscles were removed for histological or flow cytometric analysis 1–10 days after euthanasia. CTX-treated mice received 10 mg/kg TM5614, a specific PAI-1 inhibitor, orally daily, up to the day before analysis. In a separate experiment, 1 × 10^6^ CCR2^+^Ly6C^+^ BM-derived macrophages from the wild-type (WT) or PAI-1 KO mice were transplanted intravenously into PAI-1 KO recipient mice on days 0, 2, and 4 after the CTX-induced muscle injury. For histologic assessment of damaged muscle tissue post-injury, 150 μL 20 mg/kg Evans blue dye (EBD; FujiFilm Wako Pure Chemical) was injected intraperitoneally into mice 16–20 h before TA muscle sampling after euthanasia (The original unprocessed images of EBD-stained TA muscles are shown in Supplementary Fig. S3). For the performed flow cytometric analysis, TA muscle was minced with surgical scissors and dissociated with 1 mg/mL collagenase type I (FujiFilm Wako Pure Chemical) and 100 μg/mL DNase I (Roche, Basel, Switzerland) at 37 °C for 1 h, then filtered through a 40 μm mesh. Sample sizes were selected based on prior experience with similar experimental models, aiming to achieve statistical significance while minimizing animal use in accordance with the 3Rs principle. The mice were randomly assigned to experimental groups without blinding the researchers, and no animals were excluded from the analyzes.

### RNA extraction and real-time quantitative polymerase chain reaction

Total RNA was extracted from TA muscle cells using ISOGEN II reagent (Nippon Gene, Tokyo, Japan). The RNA quality was assessed using the RNA Integrity Number (RIN), and only samples with a RIN of 7 or higher were used for real-time qPCR. The RNA (0.5 μg) was reverse-transcribed to cDNA using the PrimeScript^TM^ RT-PCR kit (Takara Bio, Shiga, Japan), and gene expression levels were measured by qPCR analysis with TaqMan^TM^ Fast Advanced Master Mix and QuantStudio^TM^ 3 Real Time PCR System (Thermo Fisher Scientific). Specific TaqMan ^TM^ primers/probes (Thermo Fisher Scientific) were used: mouse *PAI-1* (Mm00435858_m1), *TNFα* (Mm00443258_m1), *IFNγ* (Mm01168134_m1), *IL-1β* (Mm00434228_m1), *IL-10* (Mm012883386_m1), *CCL2* (Mm00441242_m1), and *18S RNA* (Hs99999901_s1). The expression levels of the target genes were determined relative to that of the endogenous 18S RNA. Quantification was performed using the comparative threshold cycle (CT) method, calculated as the 2^−ΔCT^.

### Immunohistochemistry

The mice were transcardially perfused with 4% paraformaldehyde (PFA) under isoflurane anesthesia. The TA muscles were dissected, post-fixed in 4% PFA overnight at 4 °C, cryoprotected in 30% sucrose overnight, embedded in OCT compound (Section-Lab, Yokohama, Japan), and snap-frozen in liquid nitrogen. The frozen tissues were sectioned at 5 μm thickness using a cryostat (CM3050S; Leica, Wetzlar, Germany). The cryosections were stained with hematoxylin–eosin (H&E) or processed for immunofluorescence. For immunostaining, cryosections were permeabilized in chilled 80% ethanol, blocked with 5% BSA for 30 min at room temperature (approximately, 22 to 24 °C), and incubated with Alexa Fluor® 594-conjugated cleaved Caspase-3 (Cell Signaling Technology, MA, USA), FITC-conjugated anti-Laminin (Abcam, Cambridge, UK) or PE-conjugated anti-Ly6C (BioLegend, San Diego, CA, USA) for direct staining. For PAI-1 detection, sections were incubated with rabbit anti-PAI-1 primary antibodies (Abcam), followed by Alexa Fluor 488-conjugated goat anti-rabbit IgG secondary antibody (Thermo Fisher Scientific). All sections were counterstained with 4’,6-diamidino-2-phenylindole (DAPI) to visualize nuclei. Fluorescence images were obtained using HS All-in-one Fluorescence Microscope Biorevo BZ-9000 and analyzed using BZ II analyzer software (both from Keyence, Osaka, Japan).

### Flow cytometry

Flow cytometry was performed on a FACSLyric^TM^ instrument using the FACSuite^TM^ software (BD Biosciences, Franklin Lakes, NJ, USA). Data analysis was performed using FlowJo® software (Tree Star, Ashland, OR, USA). The proportion of the designated cell fraction was determined by collecting 100,000 events while excluding dead cells stained with Propidium Iodide (PI) from the data collection. The cells were then treated with an Fcγ receptor-blocking antibody (anti-mouse CD16/32). The following antibodies were used to identify macrophages: APC/Cy7-conjugated anti-mouse CD45, Brilliant Violet 421-conjugated anti-mouse/human CD11b, FITC-conjugated Ly6G, NK1.1, B220, CD3e, Siglec F, PE/Cy7-conjugated Ly6C, Alexa Fluor 700-conjugated anti-mouse F4/80, PE-conjugated CCR2 (CD192) (all from BioLegend). Cytofix/Cytoperm^TM^ buffer (BD Biosciences) was used to stain intracellular PAI-1 according to the manufacturer’s instructions. The following antibodies were used: rabbit anti-PAI-1, rabbit anti-CRT (both from Abcam, Cambridge, UK), and PE-conjugated anti-rabbit antibody. The corresponding isotype-matched antibodies were used to determine the baseline staining for the analyzes.

### Enzyme-linked immunosorbent assays

CCR2^+^Ly6C⁺ and CCR2⁻Ly6C⁻ macrophages were isolated from CTX- or PBS-treated TA muscles using a cell sorter (FACSAriaIII; BD Biosciences, San Jose, CA, USA). The cells were gated on CD45⁺CD11b⁺CD11c⁻Ly6G⁻NK1.1⁻B220⁻CD3⁻SiglecF⁻PI⁻ in order to exclude non-macrophage populations and dead cells, and subsequently sorted based on CCR2 and Ly6C expression. The sorted cells (1 × 10^5^ cells per well) were seeded into 24-well plates and cultured for 2 days in RPMI-1640 medium supplemented with 10% FBS, 2 mM L-glutamine, and antibiotics (100 μ/mL penicillin and 100 μg/mL streptomycin) at 37 °C in a humidified atmosphere containing 5% CO₂. In a separate experiment, cells were cultured for 2 days in the same medium supplemented with lipopolysaccharide (LPS; 50 ng/mL, Sigma-Aldrich, MO, USA) and interferon-gamma (IFN-γ; 20 ng/mL, Thermo Fisher Scientific). Concentrations of PAI-1, TNF-α, and IL-6 in the culture supernatants were measured using a mouse PAI-1 ELISA kit (Abcam, Cambridge, UK), mouse TNF-α ELISA kit (R&D Systems, MN, USA), and mouse IL-6 ELISA kit (R&D Systems), respectively, according to the manufacturer’s instructions.

### Efferocytosis assay

The spleen was aseptically removed from euthanized mice, transferred to a dish containing PEB, and minced into small fragments using O-ring tweezer and surgical scissors within a 40 μm nylon cell strainer. The resulting cell suspension was incubated for 5 min in ACK lysis buffer and centrifuged at 300 × *g*. The splenocytes were then incubated for 16 h in RPMI-1640 culture medium supplemented with 4 μM staurosporine (STS; FujiFilm Wako Pure Chemical) to induce apoptosis. Flow cytometric analysis using Annexin V and PI staining confirmed that more than 80% splenocytes were Annexin V^+^. Apoptotic splenocytes (3 × 10^5^ cells) were labeled with 1 μM CellTrace^TM^ Yellow dye (Thermo Fisher Scientific) and co-cultured with 3 × 10^4^ J774.1-GFP cells or CCR2^+^Ly6C^+^ macrophages labeled with 1 μM CellTrace^TM^ CFSE dye (Thermo Fisher Scientific) for 2 h at 37 °C, in various combination with or without 100 nM recombinant PAI-1 (rPAI-1; BioLegend), TM5614, and receptor-associated protein (RAP, an LRP-1 antagonist; Molecular Innovations, Novi, MI, USA). Efferocytosis was measured using flow cytometry to assess the J774.1-GFP cells or CCR2^+^Ly6C^+^ macrophages that engulfed CellTrace^TM^ Yellow dye-labeled apoptotic splenocytes. CFSE-labeled macrophages (green; excitation: 492 nm, emission: 517 nm) and yellow dye-labeled dead cells (red/yellow; excitation: 561 nm, emission: 585 nm) were observed using a BIOREVO BZ-9000 fluorescence microscope (KEYENCE) with 488 nm and 564 nm filters, respectively.

To assess efferocytosis in vivo, 100 μL TRITC-labeled dextran 2000 kDa (4 mg/kg; Thermo Fisher Scientific) was injected intraperitoneally into CTX-treated mice 3 h before TA muscle sampling, as described previously [56, 57]. Efferocytosis was measured by flow cytometry to assess TA muscle-infiltrating CCR2^+^Ly6C^+^ macrophages engulfed TRITC-labeled dextran.

### Competitive binding assay to LRP1

*N*-hydroxy-succinimidyl (NHS) ester–fluorescein dye (Thermo Fisher Scientific) was dissolved in dimethyl sulfoxide. A total of 24.65 μg human recombinant CRT (rCRT; BioLegend) was incubated with 50-fold excess NHS–fluorescein dye at room temperature (approximately 22 to 24 °C) for 1 h to facilitate conjugation. To assess the inhibitory effect of rPAI-1 on the binding of NHS-labeled rCRT to LRP1, approximately 1 × 10^5^ J774.1 cells in 100 μL RPMI-1640 medium were preincubated with 0–250 nM rPAI-1 for 15 min at 37 °C. Then, 500 nM NHS-labeled rCRT was added and incubated for another 15 min at 37 °C. The J774.1 cells were washed with RPMI-1640 medium to remove the unbound NHS-labeled rCRT. The cells were analyzed by flow cytometry to determine the percentage of cells exhibiting fluorescence, which indicates NHS-labeled rCRT binding to LRP-1. BC_50_ and IC_50_ values were determined using a four-parameter nonlinear regression model. Pearson’s linear correlation analysis was performed to assess the correlation between mean fluorescence intensity of rPAI-1 and rCRT binding to LRP1. The dissociation constant (*K*_D_) was determined using the Cheng–Prussoff equation.

$${K}_{D}=\frac{{{BC}}_{50}}{1+\frac{\left[S\right]}{{K}_{m}}}$$

where [*S*] is the ligand concentration, *K*_m_ is the Michaelis constant, which indicates the substrate concentration at which the reaction reaches half its maximum.

### Statistics and reproducibility

Data from a minimum of three independent experiments were combined and analyzed using and GraphPad Prism 10.0 (GraphPad Software, La Jolla, CA, USA). The number of animals (or samples) used in each experiment was determined based on previously published studies reporting similar effect sizes in comparable experimental models. No randomization or blinding was performed to allocate the experimental groups. The results were expressed as means ± standard deviation (SD) of three to five independent experiments, with *n* indicating the number of mice or samples per group. The Mann–Whitney unpaired *t*-test was used to compare two groups. One-way analysis of variance was conducted, followed by Tukey’s post-hoc test to compare the means of three or more independent groups. The normality of the data was assessed using the Kolmogorov–Smirnov test. The level of statistical significance was set at *p* < 0.05.

## Supplementary information

Supplemental Figure and Legend

Supplementary Table

## Data Availability

The materials generated for this study can be provided upon reasonable request. Any additional information required to reanalyze the data reported in this paper is available from the corresponding author contact upon request.
