# Supplementary material for: Inflammatory macrophage-derived plasminogen activator inhibitor-1 exacerbates inflammation through efferocytosis inhibition
Source: Cell Death Discov. 2026 Mar 27;12:195. doi: 10.1038/s41420-026-03076-0 (PMC13144377; doi:10.1038/s41420-026-03076-0)
Supplement: Supplementary file 1 — Supplemental Figure and Legend [file 41420_2026_3076_MOESM1_ESM.pdf]

Supplemental Figure S1

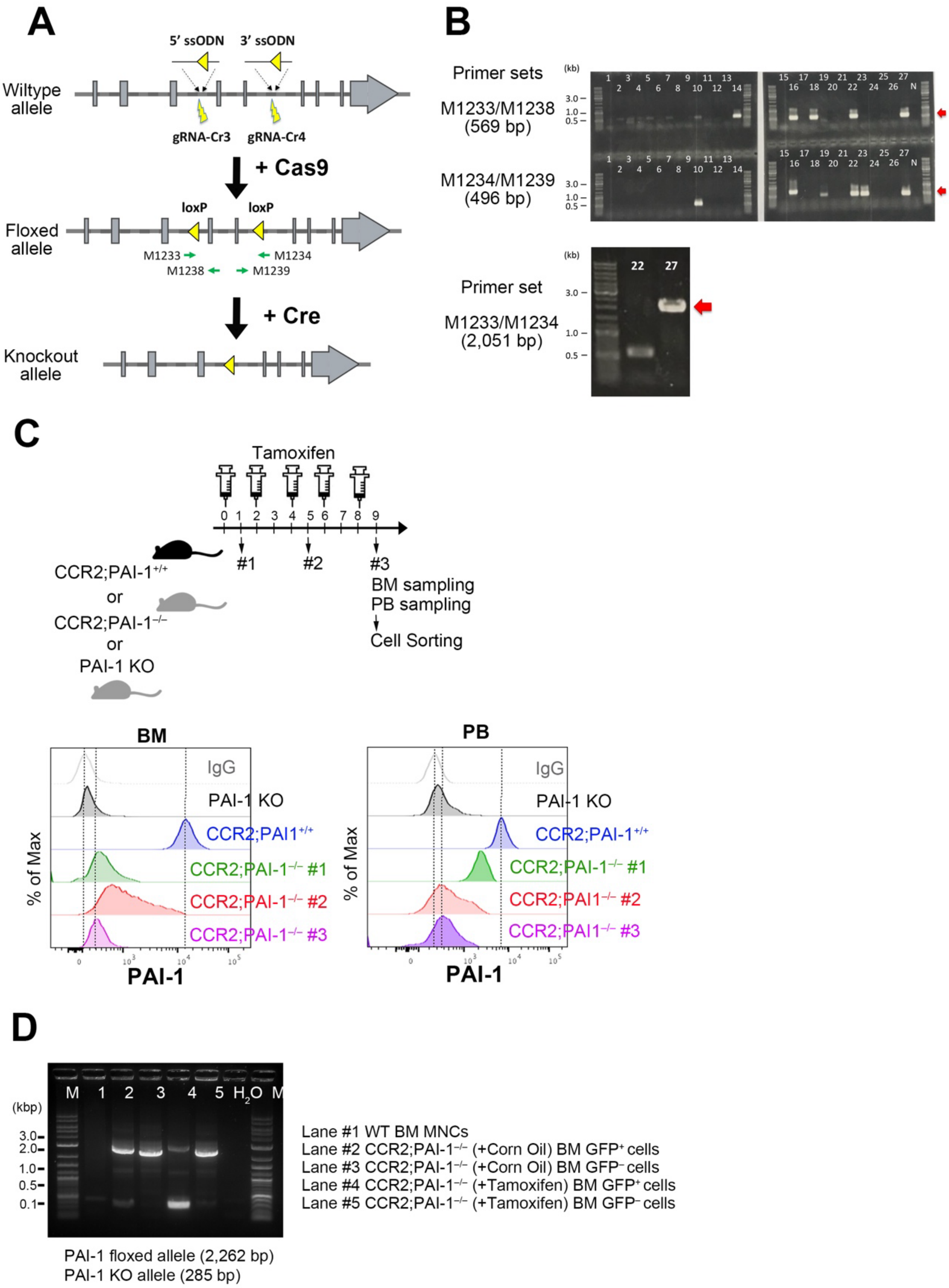

1

Supplementary Figure S1. PAI-1-floxed mouse generation and validation. (**A**) Schematic illustration of the strategy for generating a floxed allele for *Serpine1* (PAI-1) by improved-Genome editing *via* Oviductal Nucleic Acids Delivery (*i*-GONAD) method. *Serpine1* gene structures and relative positions of ssODNs (PAI1_5′_ssODN and PAI1_3′_ssODN), gRNAs (PAI1-Cr3 and PAI1-Cr4), and primers for genotyping (shown in green arrows). (**B**) Genotypes of each offspring (IDs indicated by numbers) were verified using three primer sets. The expected fragment sizes (in parenthesis) are shown under the primer set names. The PCR bands with expected size are indicated in red arrows. N: Negative control. (**C**) Verification of PAI-1 conditional knockout induction efficiency by flow cytometry. CCR2;PAI-$1^{+/+}$ mice or CCR2;PAI-$1^{-/-}$ mice were treated with tamoxifen and their peripheral blood and BM cells were collected after one, three and five doses. The GFP^+ cells ($CCR2^+$ cells) were then sorted and stained for intracellular PAI-1 before flow cytometry analysis. $CCR2^+$ cells of PAI-1 KO mice were used as a reference. IgG: isotype control. (**D**) Verification of PAI-1 conditional knockout induction efficiency by PCR. Tamoxifen or corn oil (its solvent) was administered to CCR2;PAI-$1^{+/+}$ or CCR2;PAI-$1^{-/-}$ mice five times. GFP^+ and GFP^- cells were then collected and floxed allele loss was confirmed by PCR. The original unprocessed images of Supplementary Figure S1 are shown in Supplementary Figure S2.

Supplemental Figure S2

A Supplemental Figure S1(B)

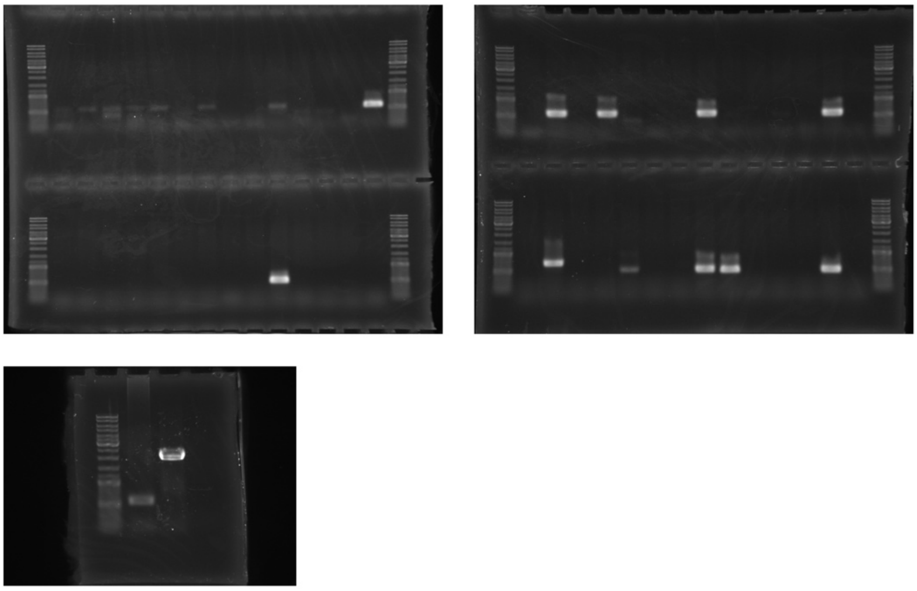

B Supplemental Figure S1(D)

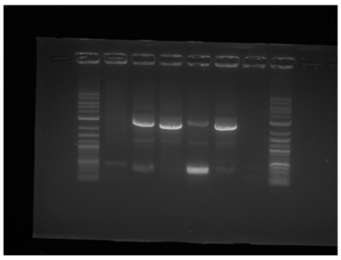

Supplementary Figure S2. Original unprocessed images of (**A**) Figure S1B and (**B**) Figure S1D

Supplemental Figure S3

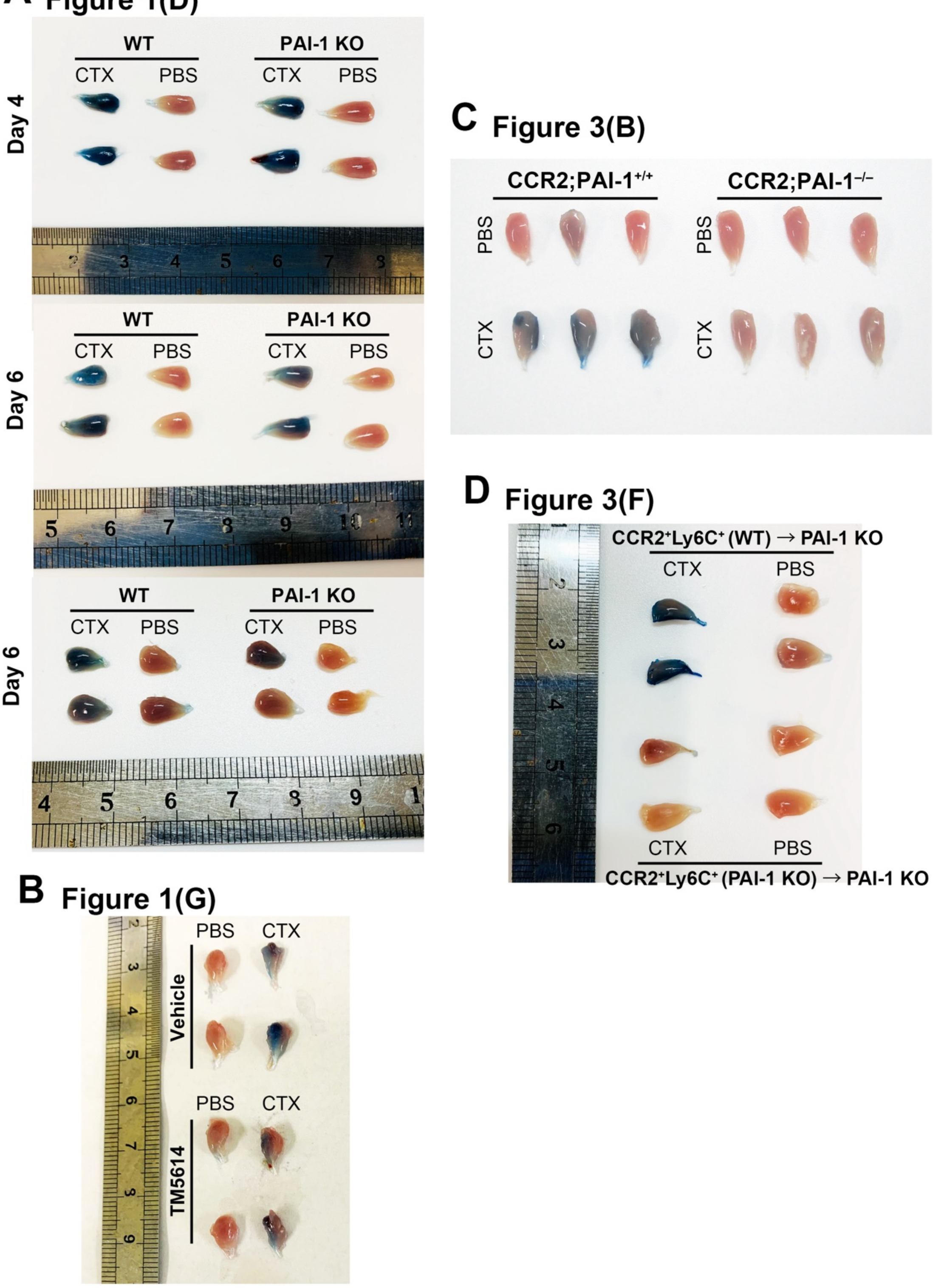

Supplementary Figure S3. Original unprocessed images of (**A**) Figure 1D, (**B**) Figure 1G, (**C**) Figure 3B, and (**D**) Figure 3F

Supplemental Figure S4

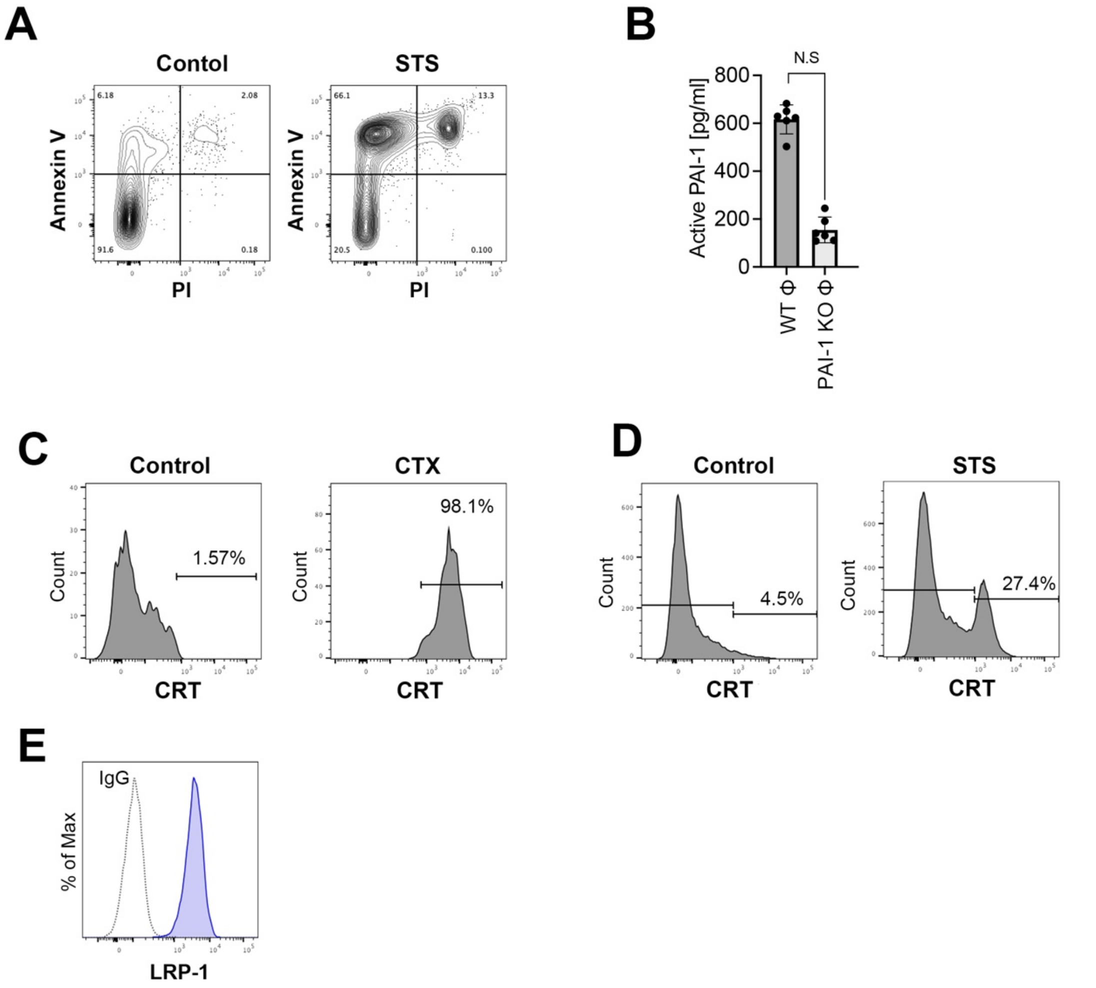

Supplementary Figure S4. Confirmation of apoptosis and efferocytosis-related antigen expression. (**A**) Representative flow cytometric profiles of Annexin V and PI of STS-treated cells. (**B**) Spontaneous active-form PAI-1 production in the $CCR2^{+}Ly6C^{+}$ macrophages isolated from WT mice (n = 6) under steady-state culture condition for 2 days, quantified by ELISA. The bars express the results as the mean ± SD. $^{**}p < 0.01$. Representative flow cytometric profiles of CRT expressed on (**C**) CTX-treated TA muscle cells and (**D**) STS-treated cells. (**E**) Representative flow cytometric profiles of LRP-1 expressed on macrophages.

Supplemental Figure S5

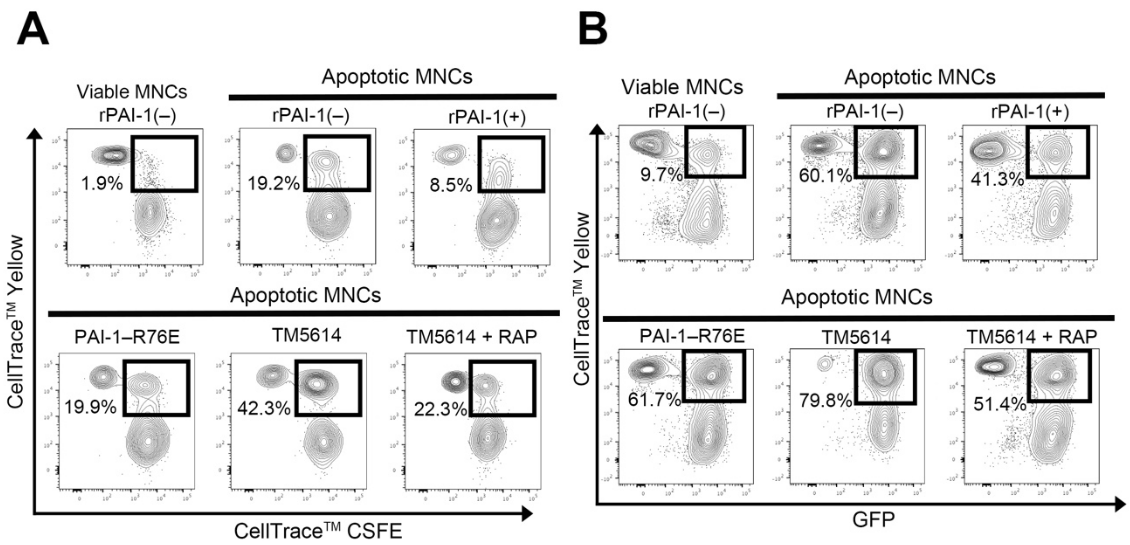

Supplementary Figure S5. Representative flow cytometric profiles on which the graphs in Figures (**A**) 6F and (**B**) 6H are based.
