# Supplementary material for: Inflammatory macrophage-derived plasminogen activator inhibitor-1 exacerbates inflammation through efferocytosis inhibition
Source: Cell Death Discov. 2026 Mar 27;12:195. doi: 10.1038/s41420-026-03076-0 (PMC13144377; doi:10.1038/s41420-026-03076-0)
Supplement: Supplementary file 2 — Supplementary Table [file 41420_2026_3076_MOESM2_ESM.docx]

**Supplementary Table S1**

| **REAGENT or RESOURCE** | **Manufacturer** | **Identifier** |
| --- | --- | --- |
| **Antibodies** | | |
| Rabbit anti-PAI-1 | Abcam, Cambridge, UK | Cat#ab66705 |
| PE anti-mouse Ly6C | Biolegend, San Diego, CA, USA | Cat#128008 clone HK1.4 |
| PE/Cyanine7 anti-mouse Ly6C | Biolegend, San Diego, CA, USA | Cat#128018 clone HK1.4 |
| Rabbit anti-Laminin | Abcam, Cambridge, UK | Cat#ab11575 |
| Biotin anti-mouse Ly6G | Biolegend, San Diego, CA, USA | Cat#127604 clone 1A8 |
| PE anti-mouse CD192 (CCR2) | Biolegend, San Diego, CA, USA | Cat#150610 clone SA203G11 |
| Alexa Fluor® 488-Cleaved Caspase-3 (Asp175) | Cell Signaling Technology, Danvers, MA, USA | Cat#9669 |
| Alexa Fluor® 594 Cleaved Caspase-3 (Asp175) | Cell Signaling Technology, Danvers, MA, USA | Cat#8172 clone D3E9 |
| Active (Cleaved) Caspase-3 | MilliporeSigma, Burlington, MA, USA | Cat#AB3623 |
| TruStain FcX^TM^ PLUS (anti-mouse CD16/32) | Biolegend, San Diego, CA, USA | Cat#156604 clone S17011E |
| APC/Cyanine7 anti-mouse CD45 | Biolegend, San Diego, CA, USA | Cat#103116 clone 30-F11 |
| Brilliant Violet 421^TM^ anti-mouse CD11b | Biolegend, San Diego, CA, USA | Cat#101236 clone M1/70 |
| FITC anti-mouse Ly6G | Biolegend, San Diego, CA, USA | Cat#127606 clone 1A8 |
| FITC anti-mouse NK1.1 | Biolegend, San Diego, CA, USA | Cat#108706 clone PK136 |
| FITC anti-mouse/human CD45R/B220 | Biolegend, San Diego, CA, USA | Cat#103206 clone RA3-6B2 |
| FITC anti-mouse CD3ε | Biolegend, San Diego, CA, USA | Cat#100306 clone 145-2C11 |
| FITC anti-mouse CD170 (Siglec F) | Biolegend, San Diego, CA, USA | Cat#155504 clone S17007L |
| Alexa Flour 700^TM^ anti-mouse F4/80 | Biolegend, San Diego, CA, USA | Cat#123130 clone BM8 |
| APC anti-mouse MHC class II | Biolegend, San Diego, CA, USA | Cat#107614 clone M5/114.15.2 |
| PE/Cy7 Annexin V | Biolegend, San Diego, CA, USA | Cat#640950 |
| Rabbit anti-LRP1 | Abcam, Cambridge, UK | Cat#ab92544 clone EPR3724 |
| Rabbit anti-Calreticulin (CRT) ER Marker | Abcam, Cambridge, UK | Cat#ab2907 |
| Cyanine5 Streptavidin | Biolegend, San Diego, CA, USA | Cat#405209 |
| PE, Donkey anti-Rabbit IgG | Biolegend, San Diego, CA, USA | Cat#406421 clone Poly4064 |
| FITC, Donkey anti-Rabbit IgG | Biolegend, San Diego, CA, USA | Cat#406403 clone Poly4064 |
| Alexa Fluor®488 goat anti rabbit IgG | ThermoFisher Scientific, Waltham, MA, USA | A-11008 |
| Alexa Fluor®594 anti rabbit IgG | ThermoFisher Scientific, Waltham, MA, USA | A-11012 |
| **Chemicals, peptides and recombinant proteins** | | |
| TM5614 (PAI-1 inhibitor) | In this paper | N/A |
| RPMI-1640 | FujiFilm Wako Pure Chemical, Osaka, Japan | Cat#189-02025 |
| Fetal Bovine Serum (FBS) | Biosera, Cholet, France | Cat#FB-1003/500  South Africa Origin |
| L-glutamine | FujiFilm Wako Pure Chemical, Osaka, Japan | Cat#073-05391 |
| Penicillin-Streptomycin | FujiFilm Wako Pure Chemical, Osaka, Japan | Cat#168-23191 |
| Tamoxifen | Sigma Aldrich, Saint Louis, MO, USA | Cat#T5648 |
| Corn oil | FujiFilm Wako Pure Chemical, Osaka, Japan | Cat#032-17016 |
| Cardiotoxin | Latoxan, Valence, France | Cat#L8102 |
| Evan Blue Dye | FujiFilm Wako Pure Chemical, Osaka, Japan | Cat#056-04061 |
| Collagenase Type-I | FujiFilm Wako Pure Chemical, Osaka, Japan | Cat#037-17603 |
| DNase I, grade II | Roche, Basel, Switzerland | Cat#10104159001 |
| TriZol^TM^ Reagent | ThermoFisher Scientific, Waltham, MA, USA | Cat#15596018 |
| ISOGEN II | Nippon Gene, Tokyo, Japan | Cat#311-07361 |
| Cytofix/Cytoperm^TM^ Fixation/Permeabilization Kit | BD Bioscience Franklin Lakes, NJ, USA | Cat#554714 |
| Dulbecco’s Phosphate buffer saline (PBS) | KAC, Hyogo, Japan | Cat#DSBN200 |
| 0.5 M Ethylenediaminetetraacetic acid (EDTA) buffer (pH 8.0) | Nippon Gene, Tokyo, Japan | Cat#311-90075 |
| 30 w/v% Albumin, Bovine Serum Albumin (BSA) | FujiFilm Wako Pure Chemical, Osaka, Japan | Cat#017-22231 |
| Monocyte Isolation Kit (BM), mouse | Miltenyi Biotec, Bergisch Gladbach, Germany | Cat#130-100-629 |
| LS Columns | Miltenyi Biotec, Bergisch Gladbach, Germany | Cat#130-042-401 |
| Staurosporine | FujiFilm Wako Pure Chemical, Osaka, Japan | Cat#197-10251 |
| PrimeScript^TM^ RT-PCR kit | Takara Bio, Shiga, Japan | Cat#RR014A |
| TaqMan^TM^ Fast Advanced Master Mix | Thermo Fisher Scientific, Waltham, MA, USA | Cat#4444557 |
| SlowFade^TM^ Gold Antifade Mountant with DAPI | Thermo Fisher Scientific, Waltham, MA, USA | Cat#S36938 |
| CellTrace^TM^ Yellow Cell Proliferation Kit | Thermo Fisher Scientific, Waltham, MA, USA | Cat#C34567 |
| CellTrace^TM^ CFSE Cell Proliferation Kit | Thermo Fisher Scientific, Waltham, MA, USA | Cat#C34554 |
| Human Receptor Associated Protein (RAP), Low Endotoxin | Melocular Innovation, Novi, MI, USA | Cat#RAP-LE |
| Tetramethhylrhodamine (TRITC) -Dextran 2,000 kDa | Thermo Fisher Scientific, Waltham, MA, USA | Cat#D7139 |
| N-Hydroxysuccinimide (NHS)-ester Fluorescein | Thermo Fisher Scientific, Waltham, MA, USA | Cat#46409 |
| Recombinant human Serpin E1 (PAI-1) protein | Biolegend, San Diego, CA, USA | Cat#753804 |
| Recombinant human calreticulin (CRT) protein | Abcam, Cambridge, UK | Cat#ab91577 |
| Recombinant Human IFN-γ, PeproTech® | Thermo Fisher Scientific, Waltham, MA, USA | Cat#300-02 |
| Lipopolysaccharide (LPS) from E.coli O111:B4, Calbiochem® | Sigma Aldrich, Saint Louis, MO, USA | Cat#437627 |
| Quantikine^TM^ ELISA Mouse TNF-α Immunoassay | R&D System, McKinley Place NE, MN, USA | Cat#MTA00B-1 |
| Quantikine^TM^ ELISA Mouse IL-6 Immunoassay | R&D System, McKinley Place NE, MN, USA | Cat#M6000B-1 |
| **Experiment models: Cells** | | |
| J774.1 | RIKEN Cell Bank, Ibaraki, Japan | Cat#RCB0434 (TKG0208); CVCL_4770 |
| Primary BM derived CCR2^+^Ly6C^+^ macrophages | This paper | N/A |
| **Experiment models: strains** | | |
| C57BL/6J | CLEA Japan, Tokyo, Japan | N/A |
| PAI-1-knockout mice (B6.129S2-Serpine1^tm1Mlg^/J) | The Jackson Laboratory, Bar Harbor, ME, USA | IMSR_JAX:002507 |
| CCR2-CreER-GFP mice [C57BL/6-Ccr2^em1(icre/ERT2)Peng^/J] | The Jackson Laboratory, Bar Harbor, ME, USA | IMSR_JAX:035229 |
| **Software** | | |
| FlowJo version 10.10 | FlowJo, LLC, Ashland, OR, USA | https://flowjo.com/flowjo/download |
| GraphPad Prism 10.2 | GraphPad Software, La Jolla, CA, USA | https://www.graphpad.com/features |
